# Neuroprotective Effects of *Glochidion zeylanicum* Leaf Extract against H_2_O_2_/Glutamate-Induced Toxicity in Cultured Neuronal Cells and Aβ-Induced Toxicity in *Caenorhabditis elegans*

**DOI:** 10.3390/biology10080800

**Published:** 2021-08-19

**Authors:** Chatrawee Duangjan, Panthakarn Rangsinth, Shaoxiong Zhang, Xiaojie Gu, Michael Wink, Tewin Tencomnao

**Affiliations:** 1 Graduate Program in Clinical Biochemistry and Molecular Medicine, Department of Clinical Chemistry, Faculty of Allied Health Sciences, Chulalongkorn University, Bangkok 10330, Thailand; duangjan@usc.edu (C.D.); panthakarn.r@chula.ac.th (P.R.); 2 Leonard Davis School of Gerontology, University of Southern California, Los Angeles, CA 90089, USA; 3 College of Horticulture, Fujian Agriculture and Forestry University, Fuzhou 350002, China; zhangshaoxiong1991@163.com; 4 Institute of Pharmacy and Molecular Biotechnology, Im Neuenheimer Feld 364, Heidelberg University, 69120 Heidelberg, Germany; Guxiaojie2003@163.com; 5 Department of Biotechnology, School of Environmental and Chemical Engineering, Dalian Jiaotong University, Dalian 116028, China; 6 Natural Products for Neuroprotection and Anti-Ageing Research Unit, Department of Clinical Chemistry, Faculty of Allied Health Sciences, Chulalongkorn University, Bangkok 10330, Thailand

**Keywords:** *Glochidion zeylanicum*, glutamate, amyloid-β, H_2_O_2_, neurite outgrowth, Nrf2/SIRT1, antioxidant, *Caenorhabditis elegans*, HT22, Neuro-2a

## Abstract

**Simple Summary:**

Antioxidants that are interrelated in the process of overcoming oxidative-stress-induced toxicity and neurite-outgrowth-inducing activity have become the main targets of neuroprotective therapy. The methanol extract of *Glochidion zeylanicum* (GZM) exhibits neuroprotective properties that are not only limited against H_2_O_2_/glutamate/Aβ insults but also promote neurite outgrowth activity. The neuroprotective effects of GZM extract were confirmed in cultured neuronal (HT-22 and Neuro-2a) cells and *C. elegans* models. To the best of our knowledge, this study is the first to report for the neuroprotective effects of GZM extract, suggesting that *G. zeylanicum* may be a neuroprotectant applicant for the prevention and alleviation of oxidative stress-induced neurodegenerative disorders, including Alzheimer’s disease. However, additional studies are required to identify the mechanistic pathways involved in neuroprotection and to confirm the efficacy of the extract in more complex model organisms.

**Abstract:**

Oxidative stress plays a crucial role in the development of age-related neurodegenerative diseases. Previously, *Glochidion zeylanicum* methanol (GZM) extract has been reported to have antioxidant and anti-aging properties. However, the effect of GZM on neuroprotection has not been reported yet; furthermore, the mechanism involved in its antioxidant properties remains unresolved. The study is aimed to demonstrate the neuroprotective properties of GZM extract and their underlying mechanisms in cultured neuronal (HT-22 and Neuro-2a) cells and *Caenorhabditis elegans* models. GZM extract exhibited protective effects against glutamate/H_2_O_2_-induced toxicity in cultured neuronal cells by suppressing the intracellular reactive oxygen species (ROS) generation and enhancing the expression of endogenous antioxidant enzymes (SODs, GPx, and GSTs). GZM extract also triggered the expression of SIRT1/Nrf2 proteins and mRNA transcription of antioxidant genes (NQO1, GCLM, and EAAT3) which are the master regulators of cellular defense against oxidative stress. Additionally, GZM extract exhibited protective effects to counteract β-amyloid (Aβ)-induced toxicity in *C. elegans* and promoted neuritogenesis properties in Neuro-2a cells. Our observations suggest that GZM leaf extract has interesting neuritogenesis and neuroprotective potential and can possibly act as potential contender for the treatment of oxidative stress-induced Alzheimer’s disease (AD) and related neurodegenerative conditions; however, this needs to be studied further in other in vivo systems.

## 1. Introduction

Alzheimer’s disease (AD) is a progressive neurologic ailment, which causes cognitive impairments and memory problems in patients [1]. The typical markers of AD histopathology are the accumulation of neurofibrillary tangles (NFTs) of hyperphosphorylated tau protein and β-amyloid (Aβ) plaques in the brain tissue, which lead to neuronal dysfunction and cell death [1]. The abnormal accumulations of Aβ and tau protein affect neuroplasticity and neurodegeneration, which correlate well with the cognitive symptoms of AD patients [1,2]. The evidence supports that oxidative stress associated neuronal cell damage plays a crucial role in the pathogenesis of AD [3]. Oxidative stress is closely associated with neurotoxicity, particularly by promoting Aβ aggregation and mitochondrial damage, which triggers neuronal cell death [1]. Glutamate is the main excitatory neurotransmitter that has been considered as an initiating factor of neuronal death in several neurodegenerative disorders [4,5]. Additionally, the high level of reactive oxygen species (ROS) accumulation is closely related to neuronal damage by glutamate [4,6,7], which occurs via receptor-mediated excitotoxicity and non-receptor-mediated oxidative toxicity [4,6].

The prevalence of AD is increasing among aging populations [1]. Currently, there are few effective drugs available for AD treatment. However, the drugs used to treat AD exert various adverse effects [1,8]. Recently, antioxidant compounds have been regarded as essential supplements for the alternative treatment and prevention of AD [9]. Prevention against oxidative-stress-induced neuronal toxicity is a key parameter in aiding neuroprotection [10]. Therefore, natural bioactive compounds found in herbs or plants with potent antioxidant and neuroprotective effects may provide complementary and alternative approaches for the treatment or prevention of AD and other neurodegenerative disorders.

*Glochidion zeylanicum* (Gaertn.) A.Juss. (family Phyllanthaceae) (GZ), native to Eastern Asia including Thailand, is a rich source of antioxidant compounds. In our previous study, the leaf extract of GZ was shown to promote oxidative stress resistance and exhibit anti-aging effects in the nematode *Caenorhabditis elegans* through the DAF-16/FoxO and SKN-1/Nrf-2 signaling pathways. [11,12]. Nevertheless, neuroprotective and neuritogenesis properties of GZ extract have not been reported.

The current study explored the neuroprotective effects of GZ extract and its underlying mechanisms on neurodegenerative events using cultured neuronal (HT-22 and Neuro-2a) cells and *C. elegans* models. We also investigated the neuritogenesis properties of GZ extract in the context of neurite outgrowth. The study provides experimental evidence of GZ extract and its applications in the prevention or treatment for neurodegenerative conditions involving oxidative stress.

## 2. Materials and Methods

### 2.1. Chemicals and Reagents

All the chemicals and reagents used in the study were purchased from Invitrogen (Carlsbad, CA, USA) and Sigma-Aldrich (St. Louis, MO, USA). Antibodies (both primary and secondary) were procured from Cell Signaling Technology (Danvers, MA, USA) (Supplementary Materials).

### 2.2. Plant Extraction

*Glochidion zeylanicum* (GZ) leaves were collected by Mrs. Laong Kwunpet and Mrs. Korakod Choosri from Jana district, Songkhla Province, in southern Thailand (7.205278° N, 100.596944° E). The plant samples were deposited for identification at the Kasin Suvatabhandhu herbarium, Department of Botany, Faculty of Science, Chulalongkorn University, Thailand (Voucher specimen No. BCU-016061). Dried and ground leaves (40 g) were extracted with methanol (400 mL) using a Soxhlet apparatus as previously described [11]. The extracts were filtrated and evaporated at 35–45 °C. The GZ methanol (GZM) extract was prepared in DMSO and stored at −20 °C as a stock.

### 2.3. Qualitative Phytochemical Screening

High-performance liquid chromatography (HPLC) of GZM extract was performed at RSU Science and Technology Research Equipment Center, Rangsit University, Thailand for the analysis of the chemical constituents (Supplementary Materials).

### 2.4. Cell Culture

Cell cultures were maintained as previously described [13]. HT-22 cells (Salk Institute, CA, USA) were cultured in Dulbecco’s Modified Eagle’s Medium (DMEM) and Neuro-2a cells (The JCRB Cell Bank, Osaka, Japan) were maintained in Ham’s Nutrient Mixture F12, supplemented with 10% fetal bovine serum and 1% streptomycin. Both cells were grown at 37 °C with 5% CO_2_ atmosphere.

### 2.5. Cell Treatment

Pre-treatment of HT-22 and Neuro-2a cells was performed with various concentrations (0.5–10 μg/mL) of GZM extract for 48 h. Glutamate or H_2_O_2_ was then mixed into the culture medium for inducing cell toxicity. For protective assays, the extract was co-treated with glutamate (HT-22:18 h, Neuro-2a: 24 h) or H_2_O_2_ (15 min). DMSO (0.1% *v/v*) served as the control group. There was no significance between DMSO treatment and untreated control (Figure S3, Supplementary Materials).

### 2.6. Determination of Cell Viability

Cell viability was determined using MTT and LDH assays. The details are provided in the Supplementary Materials.

### 2.7. Measurement of Intracellular ROS

The measurement of intracellular ROS generated upon treatment was assayed with DCFH-DA dye as previously described [11]. The details are provided in the Supplementary Materials.

### 2.8. RNA Isolation and Quantitative RT-PCR

RNA extraction was performed with TRIzol reagent. Quantitative real-time PCR was performed using standard procedures [13]. The primer sequences are listed in the Supplementary Materials. β-actin was used as the normalization control [7,13,14] (Supplementary Materials).

### 2.9. Western Blot Analysis

Proteins were obtained from Neuro-2a cells by lysis 1X using RIPA buffer containing protease inhibitor cocktail (PMSF) and quantified using Bradford assay. Briefly, protein was electrophoresed through 10% SDS polyacrylamide gel, followed by transferring to PVDF membranes and blocking (5% skim milk). The membranes were subjected to immunoblot analysis using respective antibodies (SIRT1, Nrf2, and β-actin (Supplementary Materials)). Full images of the blots are provided in the supplementary materials (Figure S2).

### 2.10. Neurite Outgrowth Analysis

Neurite outgrowth upon GZM extract was performed according to Eik et al. in Neuro-2a cells and number of neurite-bearing cells and neurite length were measured [15] (Supplementary Materials).

### 2.11. C. elegans Strains and Culture Conditions

All of the *C. elegans* strains used in the study were procured from Caenorhabditis Genetics Center (University of Minnesota, U.S.A.) and maintained with *Escherichia coli* OP50 as the food source [16]. The nematodes were cultured in NGM agar plates at 16 °C and synchronized populations were developed as described previously [7,12]. Strains used in this study include CL4176 (smg-1(cc546) I; dvIs27 [(myo-3p::A-Beta (1–42)::let-851 3′UTR) + rol-6(su1006)] X), CL2006 (dvIs2 [pCL12(unc-54/human Abeta peptide 1–42 minigene) + pRF4]), CL2355 (smg-1(cc546) dvIs50 [pCL45 (snb-1::Abeta 1–42::3′ UTR(long) + mtl-2::GFP] I), and CL2122 (dvIs15 [(pPD30.38) unc-54(vector) + (pCL26) mtl-2::GFP. The nematodes were treated with various concentrations (1.25, 2.5, and 5 μg/mL) of GZM extract with DMSO (1% *v/v*) as the control.

### 2.12. Paralysis Assay

Transgenic worms (CL4176 and CL2006) expressing human Aβ_1–42_ were used to study the effect of GZM against Aβ toxicity [17]. The transgenic worm CL4176 expressed and aggregated human Aβ_1−42_ peptides in the muscle cells after a temperature shift to 25 °C, leading to oxidative stress and paralysis [17]. The transgenic worm CL2006 constitutively express Aβ along the body-wall muscles leading to progressive paralysis in adulthood [17]. For Aβ-independent effects, CL802 strain was used as control. The worms were synchronized and treated with GZM extract at the L4 stage.

The CL4176 worms were maintained at 16 °C for 48 h and shifted to 25 °C to induce Aβ expression. The number of paralyzed worms was measured at 20, 22, 24, 26, 28, and 30 h after the shift in temperature.

The CL2006 worms were maintained at 16 °C and classified as paralyzed when they did not respond to touch or showed halo appearance around the worms’ heads. Paralyzed worms were classified and excluded from the plates every second day.

### 2.13. Chemotaxis Assay

The transgenic strains CL2122 and CL2355 were used for the identification of Aβ-induced defects in chemotaxis behavior. The transgenic worms (L4 stage) were treated with GZM extract at 16 °C for 36 h, and then shifted to 23 °C for 36 h to induce Aβ_1–42_ expression. After treatment period, plates were washed, and the nematodes were placed in the center of the plate. The attractant side contained a mixture of diacetyl (0.1% in absolute ethanol) and sodium azide (1 M) on the plate. The opposite side of the plate (control) contained a mixture of absolute ethanol and sodium azide (1 M). Worms drawn toward the attractant and repellant sides were counted, and the chemotaxis index was calculated ((the number of worms at attractant location—the number of worms at control location)/the total number of worms).

### 2.14. Statistical Analysis

The data are shown as the mean ± SEM. Data handling and statistical processing were completed using GraphPad Prism 8.0 and analyzed by one-way ANOVA, followed by Bonferroni’s test. and *p* ≤ 0.05 was considered to be significant.

## 3. Results

### 3.1. Measurement of Optimum Glutamate and H_2_O_2_ Conditions in Cultured Neuronal (HT-22 and Neuro-2a) Cells

**Figure 1:**
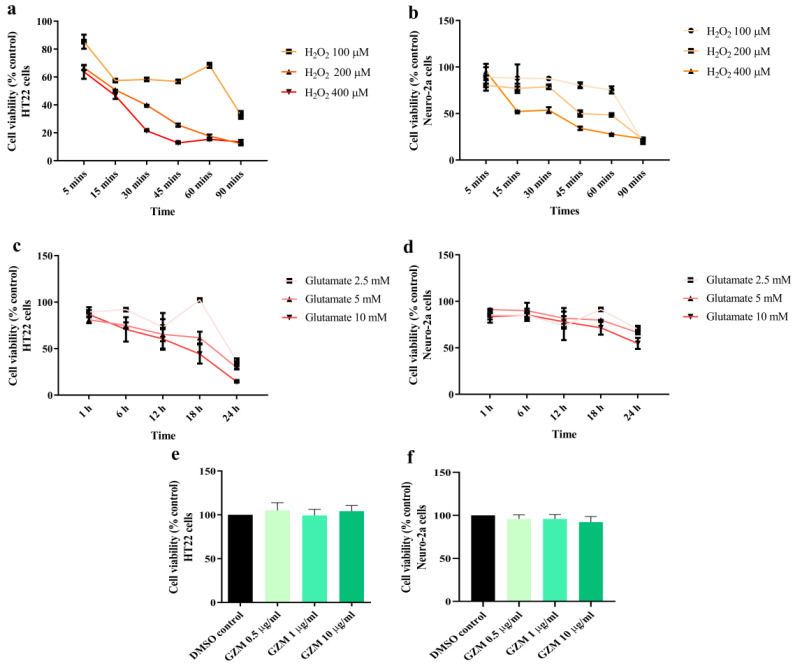
Cytotoxic effects of H_2_O_2_, glutamate, and GZM extract in neuronal (HT-22 and Neuro-2a) cells. Cell viability was analyzed using the MTT method. The neurons were exposed to different doses of H_2_O_2_ or glutamate for various time periods in HT-22 (**a**,**c**) and Neuro-2a cells (**b**,**d**). The effects of GZM extract on the cell viability of HT-22 (**e**) and Neuro-2a (**f**) cells. The cells were treated with varying concentrations of the extract for 48 h. (*p* ≤ 0.001 compared with DMSO control). The details of statistical values are provided in Table S2 (Supplementary Materials).

Neuronal cell damage induced by oxidative stress is a major phenomenon in neurodegenerative conditions [3]. The optimum conditions at which H_2_O_2_/glutamate induces neurotoxicity in HT22 and Neuro-2a cells were investigated. Exposure of HT-22 and Neuro-2a cells to different doses of glutamate (2.5–10 mM) and H_2_O_2_ (100 to 400 μM) for 1–24 h and 5–90 min, respectively. For H_2_O_2_ treatment, we found that cell viability of both cells reduced by 50% when compared with the untreated control after treatment with 200 and 400 µM H_2_O_2_ for 15 min in HT22 and Neuro-2a cells, respectively (Figure 1a,b). In the case of glutamate treatment, reduction in cell viability by 50% was obtained at 5 and 10 mM glutamate in HT22 (18 h) and Neuro-2a cells (24 h), respectively (Figure 1c,d). Therefore, these optimum conditions were used in the following experiments.

### 3.2. Neuroprotective Effects of GZM Extract against H_2_O_2_/Glutamate-Induced Neuronal Death in Cultured Neuronal (HT-22 and Neuro-2a) Cells

The cytotoxicity of GZM extract in both the cells was investigated to examine the non-lethal concentration. GZM extract (0.5–10 μg/mL) treatment was performed for 48 h in HT22 and Neuro-2a cells. When compared with the DMSO control, GZM extract treatment (0.5–10 μg/mL) did not affect the cell viability of both the cells (Figure 1e,f). The results indicated that GZM extract is non-toxic at the tested doses (0.5–10 μg/mL). Therefore, these concentrations were used in subsequent experiments.

**Figure 2:**
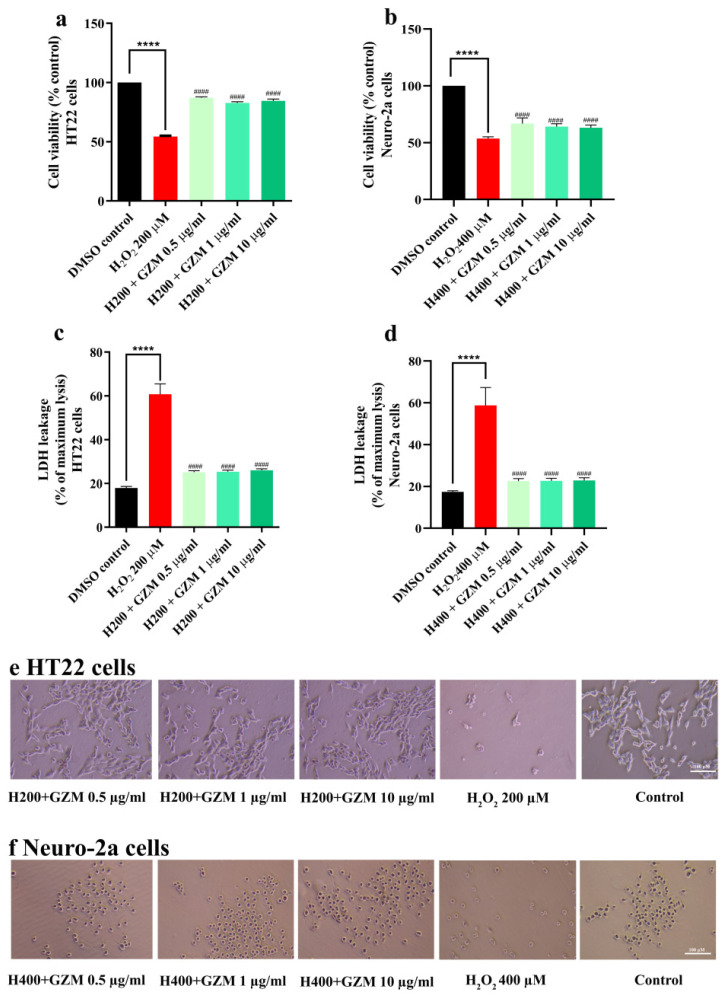
Neuroprotective effect of GZM extract against H_2_O_2_-induced toxicity in HT22 and Neuro-2a cells. MTT and LDH methods were used to analyze cell viability of HT22 (**a**,**c**) and Neuro-2a cells (**b**,**d**) after treatment with varying concentrations of GZM extract. Cell morphology of HT22 (**e**) and Neuro-2a (**f**) were observed under a light microscope (5× magnification). ((H200 and H400 corresponds to 200 and 400 µM H_2_O_2_, respectively; *n* = 3). (**** *p* < 0.0001, DMSO control vs. H_2_O_2_ treatment alone; ^####^ *p* < 0.0001, H_2_O_2_ treatment alone vs. H_2_O_2_ and GZM extract treatment. The details of statistical values are provided in Table S3 (Supplementary Materials).

The time- and dose-dependent responses of H_2_O_2_/glutamate supported and confirmed the experimental model of neurotoxicity in cultured neuronal (HT22 and Neuro-2a) cells (Figure 1a–d). Cell survival when treated with H_2_O_2_ alone was significantly lower (approximately 50%) than the DMSO control cells. However, co-treatment with GZM extract significantly reduced H_2_O_2_ cytotoxicity (Figure 2a–c,f) in both HT22 and Neuro-2a cells. This was further confirmed by the changes in the intracellular release of lactate dehydrogenase (LDH). An increase in LDH level in the culture medium is a marker of cell injury. The exposure of cells to H_2_O_2_ alone increased LDH levels (by approximately 60%). Co-treatment with GZM extract clearly attenuated the H_2_O_2_-mediated increase in LDH release (Figure 2c,d).

**Figure 3:**
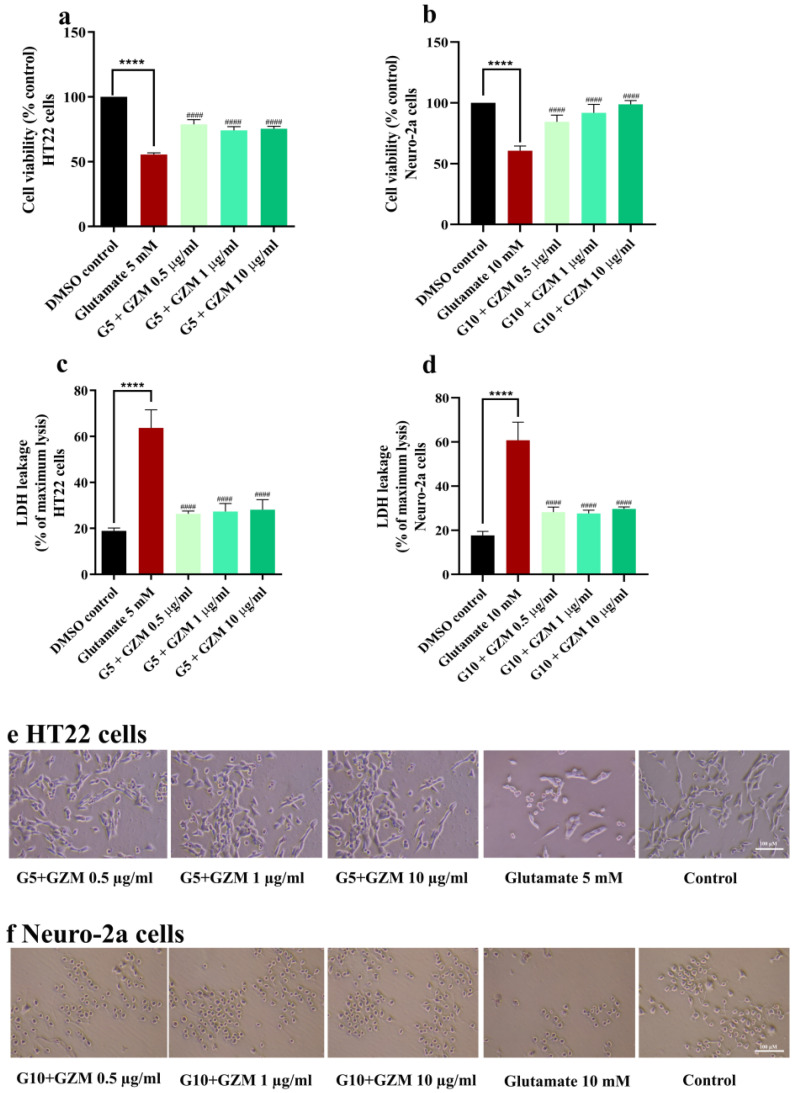
Neuroprotective effect of GZM extract against glutamate-induced toxicity in HT22 and Neuro-2a cells. MTT and LDH methods were used to analyze cell viability of HT22 (**a**,**c**) and Neuro-2a cells (**b**,**d**) after treatment with varying concentrations of GZM extract. Cell morphology of HT22 (**e**) and Neuro-2a (**f**) were observed under a light microscope (5× magnification). (G5 and G10 corresponds to 5, 10 mM glutamate; *n* = 3) **** *p* < 0.0001, DMSO control vs. glutamate treatment alone; ^####^ *p* < 0.0001 glutamate treatment alone vs. glutamate and GZM extract treatment. The details of statistical values are provided in Table S4 (Supplementary Materials).

Similarly, co-treatment with GZM extract significantly improved the cell viability compared with that of glutamate-treated cells (Figure 3a–c,f). Furthermore, glutamate-induced LDH release was also attenuated by GZM treatment (Figure 3c,d). The results suggest that GZM extract exerted an influential neuroprotective effect against H_2_O_2_/glutamate-induced neurotoxicity.

### 3.3. Neuroprotective Effects of GZM Extract against Glutamate-Induced Oxidative Stress in Cultured Neuronal (HT22 and Neuro-2a) Cells

**Figure 4:**
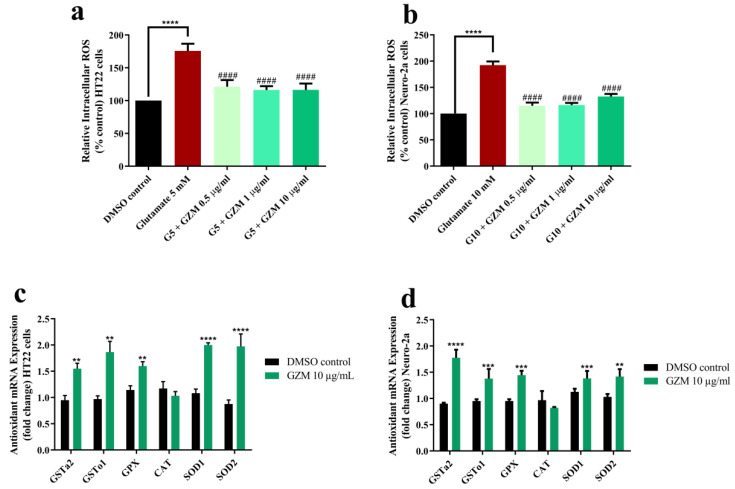
Antioxidant effects of GZM extract on glutamate-induced oxidative stress. GZM extract ameliorated ROS level in HT22 (**a**) and Neuro-2a (**b**) cells and induced the expression of antioxidant genes in HT22 (**c**) and Neuro-2a (**d**) cells (*n* = 3; ** *p* < 0.01, *** *p* < 0.001, and **** *p* < 0.0001, DMSO control vs. glutamate treatment alone; ^####^ *p* < 0.0001, glutamate treatment alone vs. glutamate and GZM extract treatment). The details of statistical values are provided in Table S5 (Supplementary Materials).

To investigate whether GZM acts against oxidative damage, intracellular ROS level was examined. Evidence of glutamate-induced toxicity in HT22 and Neuro-2a cells was shown by the increased intracellular ROS production (1.7–1.9-fold) (Figure 4a,b). Glutamate-induced production of intracellular ROS was clearly reduced by co-treatment with GZM extract (Figure 4a,b). We further investigated the endogenous antioxidant enzymes to support the protective effects of GZM extract upon glutamate exposure. The expression of antioxidant enzymes, including glutathione-S-transferase (GST), glutathione peroxidase (GPx), catalase (CAT), and superoxide dismutase (SOD) was measured.

In our previous study, the highest concentration of GZM extract (10 µg/mL) was reported to exhibit powerful antioxidant activity in vitro and in vivo [11]. In agreement with the report, the highest concentration of GZM extract (10 µg/mL) displayed potent neuroprotection in HT22 and Neuro-2a cells (Figure 2, Figure 3 and Figure 4). Further, GZM extract (10 µg/mL) increased the gene expression of antioxidant enzymes, including SOD1, SOD2, GPx, GSTo1, and GSTa2, in both HT22 and Neuro-2a cells, except for CAT (Figure 4c,d). The results indicate that GZM extract protects against glutamate/H_2_O_2_-induced cytotoxicity by the suppression of intracellular ROS production and induction of antioxidant enzyme expression.

### 3.4. Effects of GZM Extract on the Expression of SIRT1/Nrf2 Pathways

**Figure 5:**
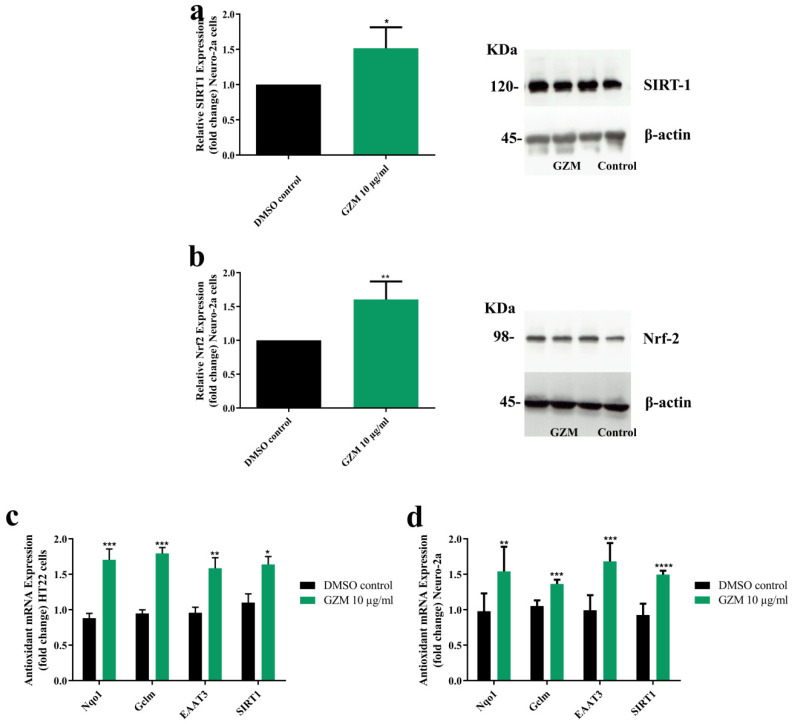
Effect of GZM extract on SIRT1/Nrf2 expression. GZM extract upregulated the expression of the SIRT1 (**a**) and Nrf2 proteins (**b**) in Neuro-2a cells. The expression levels of candidate antioxidant genes were increased after treatment with GZM extract in HT22 (**c**) and Neuro-2a (**d**) cells. (*n* = 3; * *p* < 0.05, ** *p* < 0.01, *** *p* < 0.001, and **** *p* < 0.0001, DMSO control vs. GZM extract treatment) The details of statistical values are provided in Table S6 (Supplementary Materials).

To determine the underlying mechanism of neuroprotective effects of GZM extract, we investigated the expression levels of SIRT1 and Nrf2. We found that GZM-treated neurons significantly increased SIRT1 and Nrf2 protein levels (Figure 5a,b). Further, GZM treatment potentially modulated the SIRT1/Nrf2 signaling pathway regulated-antioxidant genes NQO1, GCLM, EAAT3, and SIRT1 (Figure 5c,d). The findings indicated that GZM extract augments SIRT1/Nrf2 signaling pathway to promote cellular defenses against toxic insults.

### 3.5. Effects of GZM Extract on Neurite Outgrowth Activity in Neuro-2a Cells

**Figure 6:**
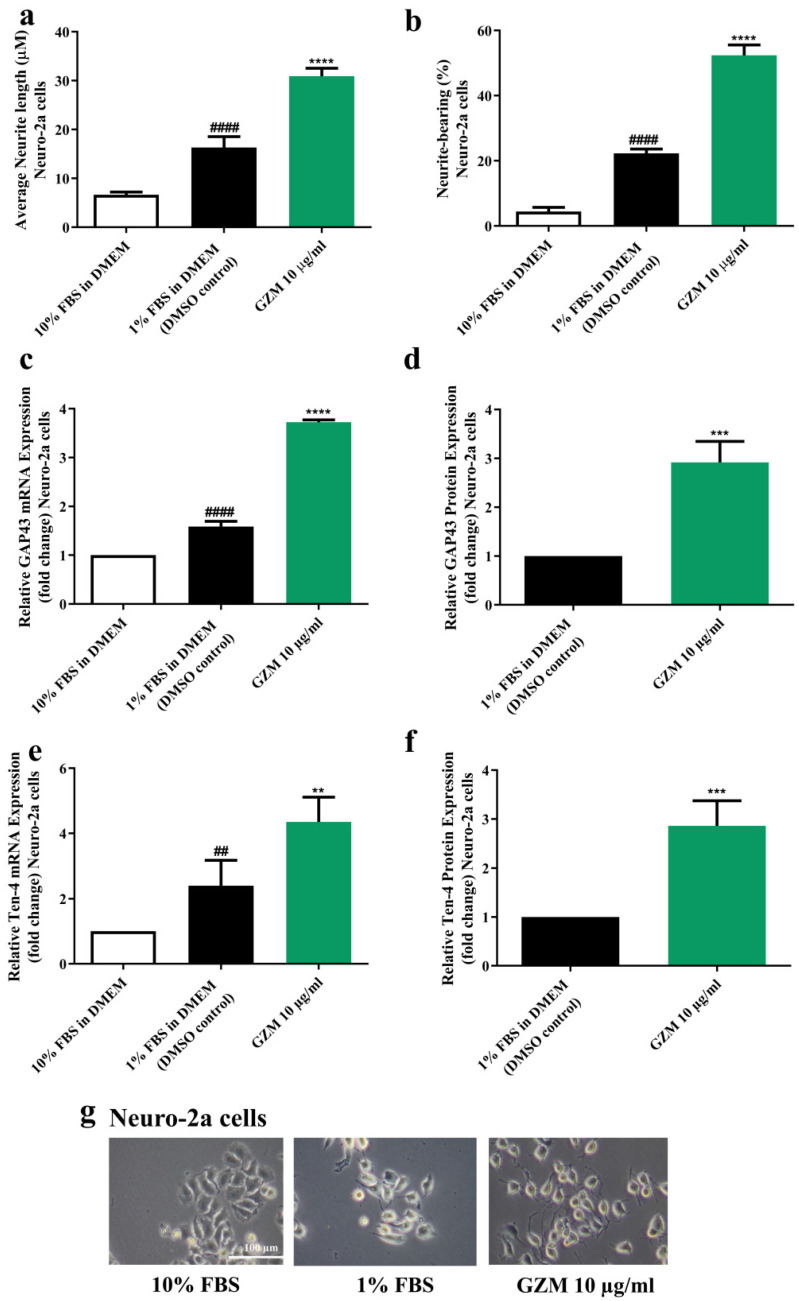
Effect of GZM extract on neurite outgrowth. GZM extract-treated Neuro-2a cells increased the average neurite length (**a**) and the percentage of neurite-bearing cells (**b**). The morphology of Neuro-2a cells was observed under a light microscope at 10× magnification (**g**). GZM extract increased the expression levels of the neuron markers GAP-43 (**c**,**d**) and Ten-4 (**e**,**f**) in Neuro-2a cells. The details of statistical values are provided in Table S7 (Supplementary Materials). (*n* = 3; ** *p* < 0.01, *** *p* < 0.001, and **** *p* < 0.0001 1% FBS (DMEM) control vs. GZM extract treatment; ^##^ *p* < 0.001 and ^####^ *p* < 0.0001 10% FBS (DMEM) vs. 1% FBS (DMEM).

To study neuronal differentiation, Neuro-2a cells were used as representatives [18]. In accordance with previous studies, serum deprivation (DMEM supplemented with 1% FBS) induced neurite outgrowth in Neuro-2a [18]. Neuro-2a cells treated with GZM extract resulted in the enhancement of neuritogenesis. GZM extract-treated Neuro-2a cells increased neurite lengths (30.92 µm) and the number of neurite-bearing cells (52.35%) when compared with the control (1% FBS) (neurite length, 16.30 µm; neurite bearing cells, 22.26%) (Figure 6a,b,g).

To further ensure the occurrence of neurite outgrowth activities, the expression of the neurite outgrowth markers, growth-associated protein 43 (GAP-43) [19], and Teneurin-4 (Ten-4) [20] were determined. GZM extract significantly upregulated GAP-43 and Ten-4 expression both at mRNA and protein levels in Neuro-2a cells when compared with the DMSO control (1% FBS) (Figure 6c–f) suggesting the neuritogenesis effect of GZM extract.

### 3.6. Neuroprotective Effects of GZM Extract against Aβ-Induced Paralysis in C. elegans

In order to further examine the neuroprotective effects of GZM extract in vivo, transgenic strains of *C. elegans* expressing Aβ were used. We first investigated the effects of GZM extract against Aβ-induced paralysis on CL4176 and CL2006 transgenic worms.

**Figure 7:**
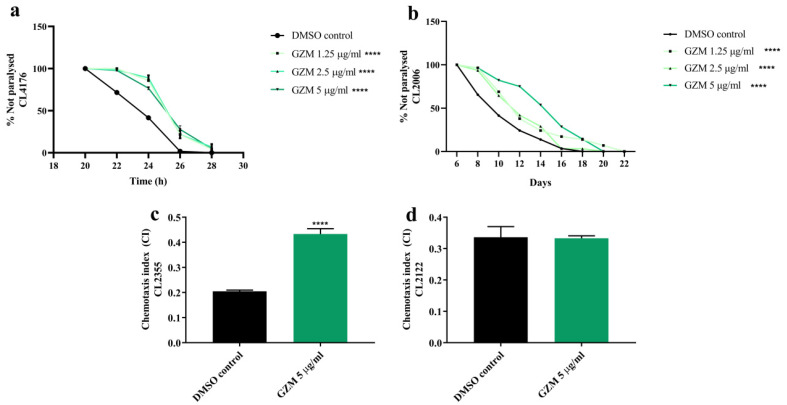
Neuroprotective effect of GZM extract against Aβ-induced toxicity in *C. elegans*. GZM extract delayed the progression of body paralysis in CL4176 (**a**) and CL2006 (**b**) worms. GZM extract increased the chemotaxis index in CL2355 (**c**) nematodes. The CL2122 strain serves as control strain (**d**). The represented times are from the instigation of temperature increase. The details of statistical values are provided in Table S8 (Supplementary Materials). (*n* = 3); and **** *p* < 0.0001, DMSO control vs. GZM extract treatment.

In our previous study, GZM extract showed resistance toward oxidative stress and lifespan extension properties in *C. elegans* at the non-toxic concentration (1–5 µg/mL) [11]. The control strain CL802 (not expressing Aβ) exhibited no paralysis, despite treatment (data not shown) at the tested concentration (1–5 µg/mL). We found that the CL4176 worms treated with GZM extract had markedly showed a delay in PT50 (time taken for 50% of the nematodes to be paralyzed) when compared to the DMSO control (Figure 7a) (Table S1, Supplementary Materials). Similarly, GZM extract also exhibited a delay in the Aβ-induced paralysis of CL2006 transgenic worms during adulthood (Figure 7b). The results suggest that GZM extract protects *C. elegans* from Aβ-induced damage.

### 3.7. Neuroprotective Effects of GZM Extract against Aβ-Induced Defects in Chemotaxis Behavior in C. elegans

To support the neuroprotective effects of GZM extract in *C. elegans*, chemotaxis assay with CL2122 and CL2355 transgenic worms was conducted. Transgenic CL2355 worms were found to express Aβ_1–42_ in the neuronal cells, which resulted in defects in chemotaxis sensitivity [21]. Since the GZM extract at the concentration 1–5 μg/mL delayed paralysis in CL4176 transgenic worms, 5 μg/mL GZM extract was chosen for all ensuing experiments. To determine the potential effect of GZM extract on chemotaxis behavior, we used diacetyl as an attractant. The results are expressed as the chemotaxis index compared with that of the untreated control. First, we found that the GZM extract treatment did not affect the chemotaxis behavior of the CL2122 mutant (transgenic control strain) (Figure 7d). CL2355 worms treated with GZM extract showed a significantly improved chemosensory response to the attractant (diacetyl) compared to the untreated control (Figure 7c), indicating that GZM extract has the potential to improve chemotaxis behavior in *C. elegans* against Aβ. Taken together, the effect of GZM extract on Aβ-dependent behavior of *C. elegans* may provide the opportunity to decipher GZM-extract-mediated neuroprotection against AD.

## 4. Discussion

Alzheimer’s disease (AD) is a neurodegenerative condition prevalently found in elderly populations worldwide [1]. At present, there is no satisfactory treatment for the broad range of pathological conditions associated with AD. Oxidative stress is intensively correlated with neurodegenerative diseases, particularly AD [3]. Natural products containing potent antioxidants may be used as alternative treatments or for the prevention of neurodegenerative diseases. In the current study, we explored the effects of GZM extract on neurodegeneration. This is the first report to describe the neuroprotective effects of GZ leaf extract in vitro (cultured neuronal cells HT22 and Neuro-2a) and in vivo (*C. elegans*).

Oxidative stress-induced neuronal toxicity is considered as one of the main factors allied to the development of neurodegenerative diseases, including AD [3]. High levels of glutamate activate the production of ROS, leading to neurotoxicity, neuronal cell damage, and eventually, neuronal cell death [4,6]. In addition, hydrogen peroxide (H_2_O_2_) is a common essential mediator of oxidative stress in neuronal cells [22]. Therefore, glutamate and H_2_O_2_ were used as the neurotoxic agents to induce neuronal cell death in this study. To study the neuroprotective effect against glutamate toxicity, mouse hippocampal neuronal HT22 cells lacking the ionotropic glutamate receptors were employed [6]. In addition, mouse neuroblastoma Neuro-2a cells are widely used as representatives in the study of neurite outgrowth and neuronal differentiation [23]. First, the neuroprotective effects of GZM extract against H_2_O_2_/glutamate-induced toxicity were determined using HT22 and Neuro-2a cells. We found that the GZM extract exerted a potent neuroprotective effect against glutamate/H_2_O_2_-induced cytotoxicity in both HT22 and Neuro-2a cells.

Oxidative stress upon glutamate exposure can be a reason of structural degradation, DNA damage, and mitochondria dysfunction, which play important roles in neuronal cell death [6]. In our study, the viability of cells treated with glutamate alone was remarkably low (approximately 50% lower) as opposed to that of untreated control cells. In addition, a notable elevation in intracellular ROS level was detected in HT22 (1.7-fold) and Neuro-2a (1.9 fold) cells upon glutamate treatment when compared to that of the untreated control. Consequently, glutamate-induced cytotoxicity in neuronal (HT22 and Neuro-2a) cells was indeed found to be allied with an increase in intracellular ROS corroborating with the previous reports [24]. The endogenous antioxidant enzymes, CAT, SOD, GST, and GPx play essential roles in neuroprotection by preventing ROS-mediated cellular damage [25,26,27]. We found that the GZM extract can counteract H_2_O_2_/glutamate-induced cytotoxicity by suppressing intracellular ROS production and augmenting the expression of antioxidant genes corresponding to the previous report, in which GZ extracts stimulated the expression of *Sod-3* and *Gst-4* in *C. elegans* [11].

The transcription factor NRF2 (nuclear factor erythroid 2-related factor 2) is the main cellular mechanism response to oxidative stress and cell damage [28,29,30,31]. The SIRT1/Nrf2 signaling pathway is an important signaling pathway to balance oxidative stress, which is actively involved in various neurodegenerative diseases [25,26]. SIRT1 manages transcription factors, inclusive of Nrf2, which acts as a master regulator of the antioxidant defense system. Nrf2 binds to the antioxidant response element (ARE), leading to enhanced expression of detoxifying enzymes (phase II) (GPx, GSTo1, and GSTa2) and antioxidative genes (SOD, CAT, NQO1, GCLM, and EAAT3) [25,26]. In accordance with reports, expression of SIRT1 and Nrf2 proteins as well as antioxidant genes, including NQO1, GCLM, EAAT3, and SIRT1, were upregulated upon GZM treatment. Further, by endogenous antioxidant enzyme expression, SOD, GPx, GSTo1, and GSTa2 were also significantly increased, which might be due to the activation of Nrf2/ARE. The results are in agreement with our previous study, in which GZ leaf extracts were demonstrated to provide oxidative stress resistance properties in *C. elegans* through SKN-1/Nrf-2 dependent mechanisms [11]. Collectively, our findings suggest that the antioxidant defense properties of GZM extract in cultured neuronal (HT22 and Neuro-2a) cells involve SIRT1/Nrf2 signaling pathway. NRF2 has been shown to influence the direction of glutamine derived glutamate metabolism, including the generation of antioxidant glutathione (GSH) for maintaining redox homeostasis [13]. In this study, we first focus on the antioxidant properties of GZM extract through the SIRT1/Nrf2 signaling pathway, which is a well-known antioxidant defense. We found that GZM extract augments SIRT1/Nrf2 signaling pathway to promote cellular defense against toxic insults. Nevertheless, the investigation of the effects of GZM extract on the SIRT1/Nrf2 signaling pathway under glutamate-induced toxicity conditions are interesting topics to confirm the antioxidant properties of GZM extract through the SIRT1/Nrf2 signaling pathway.

The imbalances between free radicals and antioxidants (oxidative stress) have been implicated in the progression of neurodegenerative diseases [1,3]. During the last decade, polyphenolic compounds have been explored extensively in neuroprotective effects because of their antioxidant properties [13,32,33,34,35]. Moreover, indirect antioxidant compounds [36] (moderate antioxidant systems and related pathways, but not direct interactions with reactive species) such as docosahexaenoic acid (DHA) [37], Vitamin K [38], and sinapic acid [39] have emerged in alternative treatment of neurodegenerative diseases. In view of the above, our findings are supported in that the beneficial effects of GZM on neuroprotective effects are possibly dependent on its antioxidant activity.

Neuritogenesis or neurite outgrowth plays an essential role in neuronal development [32]. The neurite outgrowth properties of GZM extract were determined using Neuro-2a cells. GZM extract was shown to exert neurite outgrowth activities as it elevated neurite length and the number of neurite-bearing cells. In addition, these phenomena were further confirmed by the increased expression of neurite outgrowth markers GAP-43 and Ten-4. Several studies have reported that polyphenols, including gallic acid [32,34], catechin [13,40,41] and quercetin [33,42,43], promote neurite outgrowth activity. The presence of phenolic compounds as well as gallic acid, catechin, and quercetin in GZM extract may be responsible for the observed behavior (Supplementary Figure S1) [11,12].

The Aβ plaques and tau proteins are well-known hallmarks of AD [44,45]. Abnormal Aβ production and deposition play important roles in the pathogenesis of AD [44,46]. *C. elegans* is a useful model organism for understanding age-associated neurodegeneration [47]. This worm has a basic nervous system that only contains 302 neurons that comprehensively map the neuronal connectivity [47]. Moreover, the transgenic worm exhibits the accumulation of protein carbonyls and ROS, similar to the pathology of AD [48]. Thus, we leveraged the established correlation between Aβ expression and apparent symptoms, including paralysis and chemotaxis behavior, in transgenic *C. elegans* model. The paralysis phenotype in Aβ expressing (in muscles) strains CL4176 and CL2006 was first investigated. GZM extract deferred Aβ-induced onset and aged paralysis in transgenic CL4176 and CL2006 worms, respectively. To connect Aβ toxicity with neurological functions, the chemotaxis behavior of Aβ expressing strain (in neurons) CL2355 was explored. GZM extract was found to exhibit improvements in the chemotaxis behavioral defects in transgenic CL2355 worms against Aβ. Collectively, these results indicate that GZM extract protects muscular and neurological functions in *C elegans* from Aβ-induced toxicity. Diverse studies have reported that the transcription factors DAF-16 and SKN-1 play crucial roles in Aβ deposition in the nematodes [47,48]. In our previous studies, we proved that the GZM leaf extract can stimulate oxidative stress resistance via the DAF-16/FoxO and SKN-1/Nrf-2 signaling pathways, leading to improvements in the lifespan and health of *C. elegans* [11]. We speculate that DAF-16/FoxO and SKN-1/Nrf-2 may play roles in GZM extract-mediated protection against Aβ toxicity.

Several studies have reported that phenolic antioxidants can protect against neurotoxicity induced by glutamate, Aβ peptides, and oxidative stress, which mainly depends on Nrf2/ARE signaling pathways [13,17,49,50,51,52]. GZM extract contains phenolic compounds including gallic acid, catechin, and quercetin (Supplementary Figure S1) [11,12]. Thus, neuroprotective effects mediated by the SIRT1/Nrf2 signaling pathway may result from the presence of bioactive compounds in the GZM extract. Further studies are needed to confirm the involvement of neuroprotective effects and explore more possible targets of the GZM extract.

## 5. Conclusions

In conclusion, neuroprotective effects of the GZM extract against H_2_O_2_/glutamate/Aβ-induced toxicity and neurite outgrowth properties were demonstrated in this study. GZM extract protects against H_2_O_2_/glutamate-induced oxidative toxicity by inhibiting the accumulation of intracellular ROS and increasing endogenous antioxidant enzymes via the SIRT1/Nrf2 signaling pathway. Moreover, GZM extract protects against Aβ-induced toxicity in *C. elegans*. In addition to its neuroprotective effects, GZM extract exhibited beneficial effects in promoting neurite outgrowth activity. Antioxidants that are interrelated in the process of overcoming oxidative-stress-induced toxicity and neurite-outgrowth-inducing activity have become the main targets of neuroprotective therapy. GZM extract exhibits neuroprotection properties that are not only protective against H_2_O_2_/glutamate/Aβ-induced toxicity but also promote neurite outgrowth activity. The neuroprotective effects of GZM extract were successfully confirmed in cultured neuronal (HT22 and Neuro-2a) cells and *C. elegans* models. The present study confirms, for the first time, the beneficial neuroprotective effects of GZM extract, suggesting that *G. zeylanicum* may be a neuroprotectant candidate for the prevention and treatment of AD and other neurodegenerative disorders related to oxidative stress. However, further studies are required in more complex model organisms to illuminate the active components of GZM extract and the mechanistic pathways involved in neuroprotection in order to support the therapeutic potential of the plant extracts for alternative or adjunct treatment of neurodegenerative diseases.

## Data Availability

The data used to support the findings of this study are included within the article and supplementary information file.

## Supplementary Materials

The following are available online at https://www.mdpi.com/article/10.3390/biology10080800/s1, Supplementary Materials and Methods: 1.1 Chemicals and reagents, 1.2 Qualitative phytochemical screening, 1.3 Determination of cell viability, 1.4 Measurement of intracellular ROS, 1.5 RNA isolation and quantitative RT-PCR, 1.6 Western blot analysis, 1.7 Measurement of neurite outgrowth and neurite-bearing cells. Supplementary Figures and Tables: Figure S1. HPLC chromatograms of GZM extract, Figure S2. The full images of electrophoretic blots by WB analysis in Neuro-2a cells, Figure S3. The comparison between untreated control and DMSO (0.1% v/v) control, Table S1. PT50 values for the treatments in paralysis assay, Table S2. The summary of statistical values of glutamate, H2O2, and GZM treatment in neuronal (HT-22 and Neuro-2a) cells, Table S3. The summary of statistical values of H2O2 and GZM extract treatment in neuronal (HT-22 and Neuro-2a) cells, Table S4. The summary of statistical values of glutamate and GZM extract treatment in neuronal (HT-22 and Neuro-2a) cells, Table S5. The summary of statistical values of intracellular ROS levels and antioxidant enzyme genes expression after GZM extract treatment in neuronal (HT-22 and Neuro-2a) cells, Table S6. The summary of statistical values of SIRT-1 and Nrf2 expression after GZM extract treatment in Neuro-2a cells, Table S7. The summary of statistical values of neurite outgrowth after GZM extract treatment in Neuro-2a cells, Table S8. The summary of statistical values of neuroprotective effect after GZM extract treatment in *C. elegans.*

## Abbreviations

AD: Alzheimer’s disease; AREs, antioxidant response elements; CAT, catalase; DMEM, Dulbecco’s Modified Eagle’s Medium; DMSO, dimethyl sulfoxide; DNA, deoxyribonucleic acid; EAAT3, excitatory amino acid transporter 3; FBS, fetal bovine serum; GAP-43, growth associated protein 43; GCLM, glutamate cysteine ligase complex modifier subunit; GLC-MS, gas/liquid chromatography-mass spectrometry; GPx, glutathione peroxidase; GST, glutathione-S-transferase; GST-4, glutathione S-transferase 4; GZ, *Glochidion zeylanicum*.; GZM, GZ methanol extract; H_2_DCF-DA, 2,7-Dichlorofluorescein diacetate; H_2_O_2_, hydrogen peroxide; HamF12, Ham’s Nutrient Mixture F12; HPLC, high-performance liquid chromatography; LDH, lactate dehydrogenase; MTT, 3-(4,5-Dimethylthiazol-2-yl)-2,5-diphenyltetrazolium bromide; NQO1, NAD(P)H, quinone oxidoreductase 1; Nrf2, nuclear factor-E2-related factor 2; PCR, polymerase chain reaction; PBS, phosphate buffer saline; RNA, ribonucleic acid; ROS, reactive oxygen species; SIRT, Sirtuin 1; SOD, superoxide dismutase; SOD-3, superoxide dismutase-3; Ten-4, Teneurin-4.
