# Supplementary material for: Neuroprotective Effects of Glochidion zeylanicum Leaf Extract against H2O2/Glutamate-Induced Toxicity in Cultured Neuronal Cells and Aβ-Induced Toxicity in Caenorhabditis elegans
Source: Biology (Basel). 2021 Aug 19;10(8):800. doi: 10.3390/biology10080800 (PMC8389654; doi:10.3390/biology10080800)
Supplement: Supplementary file 1 [file biology-10-00800-s001.zip › biology-1311715 supplementary.pdf]

Supplementary Material

1 Supplementary Materials and Methods

1.1 Chemicals and reagents

DMSO, DMEM, FBS, and 2,3-Butanedione (Diacetyl) were obtained from Sigma-Aldrich (St. Louis, MO, USA), sodium azide was obtained from Appli-Chem (Darmstadt, Germany), H2DCFDA was obtained from Molecular Probes (Eugene, OR, USA), and MTT was obtained from Bio Basic (Markham, Ontario, Canada). The penicillin/streptomycin solution was purchased from Gibco (Waltham, MA, USA), the RIPA buffer was purchased from Abcam (Cambridge, UK), and Trizol was purchased from Invitrogen (Carlsbad, CA, USA). The CytoTox 96® kit for the LDH assay was obtained from Promega (Madison, WI, USA), and the RT PreMix and qPCR Master Mix solutions were obtained from Bioneer (Daejeon, South Korea).

The primary antibodies used for the Western blot (analysis sirtuin 1 (SIRT1), nuclear factor-E2-related factor 2 (Nrf2), and β-actin antibodies) were purchased from Cell Signaling Technology (Danvers, MA, USA), and the GAP43 antibody was purchased from Abcam (Cambridge, UK). Secondary antibodies were purchased from Cell Signaling Technology (Danvers, MA, USA).

1.2 Qualitative phytochemical screening

SHIMADZU LC-10 HPLC equipped with an analytical C18 reversed-phase column (ODS3 C18, 4.6 × 250 mm i.d., 5 μm particle size) and a UV detector (best condition at 220 nm) were used. The mobile phase consisted of 0.02 M sodium acetate buffered to a pH of 4 with 0.0125 M citric acid containing 0.042 M methanesulfonic acid and 0.1 mM EDTA. The flow rate was set at 1 mL/min. Working standard solutions were freshly prepared in 0.05 M perchloric acid containing 0.1 mM Na2EDTA on ice and stored at −20 °C before use. Peaks were identified by comparing the retention time of each peak in the sample solution, where each individual peak was further compared to the standard solution of gallic acid, catechin, and quercetin served as an internal standard.

1.3 Determination of cell viability

The MTT and LDH assays were used to measure cell viability. To assess the MTT assay, a MTT solution (final concentration 0.5 mg/mL) was added to the culture medium and incubated for 3 h at 37 °C. Then, the solution was removed and the formazan crystals were solubilized by an DMSO–ethanol mixture (1:1, v/v). The absorbance at 550 nm was measured using an EnSpire® Multimode Plate Reader (Perkin-Elmer, Waltham, MA, USA). Results are expressed as a percentage relative to the DMSO control.

To perform the LDH assay, the activity of LDH release in the culture medium was measured using the CytoTox 96® assay (Promega) in accordance with the manufacturer's instructions. The culture supernatant was incubated with a substrate mix for 30 min in the dark at RT, followed by

the addition of a stop solution. The absorbance at 490 nm was read using an EnSpire® Multimode Plate Reader (Perkin-Elmer, Waltham, MA, USA). Results are expressed as a percentage of the maximum LDH release obtained by complete cell lysis.

1.4 Measurement of intracellular ROS

HT22 and Neuro-2a cells were treated with GZM extract and washed with ice-cold PBS. Then, 10 μM H2DCFDA was added to the culture medium and incubated for 30 min at 37 °C, followed by washing with Hank's balanced salt solution (HBSS). The fluorescence intensity (excitation = 485 nm; emission = 535 nm) was measured using an EnSpire® Multimode Plate Reader (Perkin-Elmer). Data are expressed as the percentage of fluorescence intensity of the treated cells relative to that of the untreated control.

1.5 RNA isolation and quantitative RT-PCR

The total RNA was extracted using Trizol reagent and converted to cDNA using AccuPower RT PreMix (Bioneer) and oligo (dT). The cDNA of candidate genes was amplified and quantified in a SYBR Green PCR Master Mix on Exicycler™ 96 (Bioneer). The PCR conditions were as follows: 95 °C for 15 min, followed by 45–55 cycles of denaturation at 95 °C for 15 s, and primer annealing/extension at 55 °C for 30 s. A melting-curve analysis was performed to determine the primer specificity. The relative expression of each gene was normalized against the internal control gene (β-actin), and expression levels were analyzed using the 2-ΔΔCT method. The gene-specific sequences of the primers were SOD1 (forward: 5'-CAGGACCTCATTTTAATCCTCAC-3', reverse: 5'- CCCAGGTCTCCAACATGC-3'), SOD2 (forward: 5'- CTGGACAAACCTGAGCCCTA-3', reverse: 5'-TGATAGCCTCCAGCAACTCTC-3'), CAT (forward: 5'- CAGCGACCAGATGAAGCA-3', reverse: 5'- CTCCGGTGGTCAGGACAT-3'), GPx (forward: 5'-ACAGTCCACCGTGTATGCCTTC-3', reverse: 5'- CTCTTCATTCTTGCCATTCTCCTG-3'), GSTo1 (forward: 5'- CAGCGATGTCGGGAGAAT-3', reverse: 5'-GGCAGAACCTCATGCTGTAGA-3'), GSTa2 (forward: 5'- TCTGACCCCTTTCCCTCTG-3', reverse: 5'- GCTGCCAGGATGTAGGAACT-3'), NQO1 (forward: 5'-CGACAACGGTCCTTTCCAGA-3′, reverse: 5'-TCCCAGACGGTTTCCAGAC-3′), GCLM (forward: 5'-GGAGCTTCGGGACTGTATCC-3′, reverse: 5'-AACTCCAAGGACGGAGCAT-3′), EAAT3 (forward: 5'-ATGATCTCGTCCAGTTCGGC-3′, reverse: 5'-TGACGATCTGCCC AATGCTT-3′) and β-actin (forward: 5'- GGCTGTATTCCCCTCCATCG-3', and reverse: 5'-CCAGTTGGTAACAATGCCATGT-3') as the normalization control.

1.6 Western blot analysis

The protein was electrophoresed on 10% SDS polyacrylamide gel and transferred to PVDF. The membranes were blocked with 5% skimmed milk in Tris buffered saline (TBS-T, 0.1% Tween 20) and incubated overnight at 4 °C with primary antibodies specific for SIRT1 (1:2000), Nrf2 (1:8000) or β-actin (1:16,000). Subsequently, the membranes were incubated with a secondary

antibody (1:10,000) at room temperature for 60 min. Finally, the bands were visualized using a film exposure with the chemiluminescence detection system (ECL™ Select western blotting detection reagent: Sigma-Aldrich, MO, USA) and evaluated using ImageJ software (National Institutes of Health, Bethesda, MD).

1.7 Measurement of neurite outgrowth and neurite-bearing cells

The Neuro-2a cells were cultivated with a density of 15000 cells/well in 6-welled tissue culture plates, supplemented with 10% FBS medium for 12–18 h. Cells were carefully washed with PBS 3 times before treatment. Then, the cells were treated with different concentrations of AOH (0.25-1 μg/mL) and AOM (0.5–10 μg/mL) in starved conditions (1% FBS medium) for 48 h.

The Neuro-2a cells (100 cells /treatment) were photographed using a bright-field microscope under 10 × magnification. The cells were marked as differentiated if one or more neurites were longer than the diameter of the cell body. Neurite length was measured from the cell membrane of the body cell to the end of the growth cone, and the percentage of neurite-bearing cells was quantified with ImageJ software (National Institutes of Health, Bethesda, MD). The expression levels of the specific neuronal differentiation markers growth-associated protein 43 (GAP43) and Teneurin 4 (Ten-4) were investigated by real-time PCR and Western blot analysis. Cells in complete growth medium (10% FBS) were used as negative control, and cells in starved conditions (1% FBS) medium were used as the control. Cells treated with 20 μM retinoic acid acted as the positive control.

2 Supplementary Figures and Tables

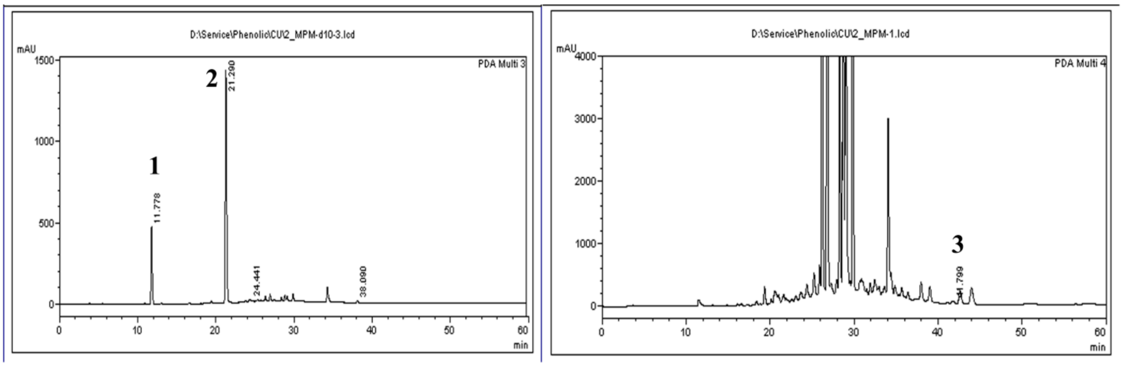

Figure S1. HPLC chromatograms of GZM extract.

The major compounds found in GZM extract including gallic acid (1), catechin (2), and quercetin (3) by HPLC at 280 and 331 nm.

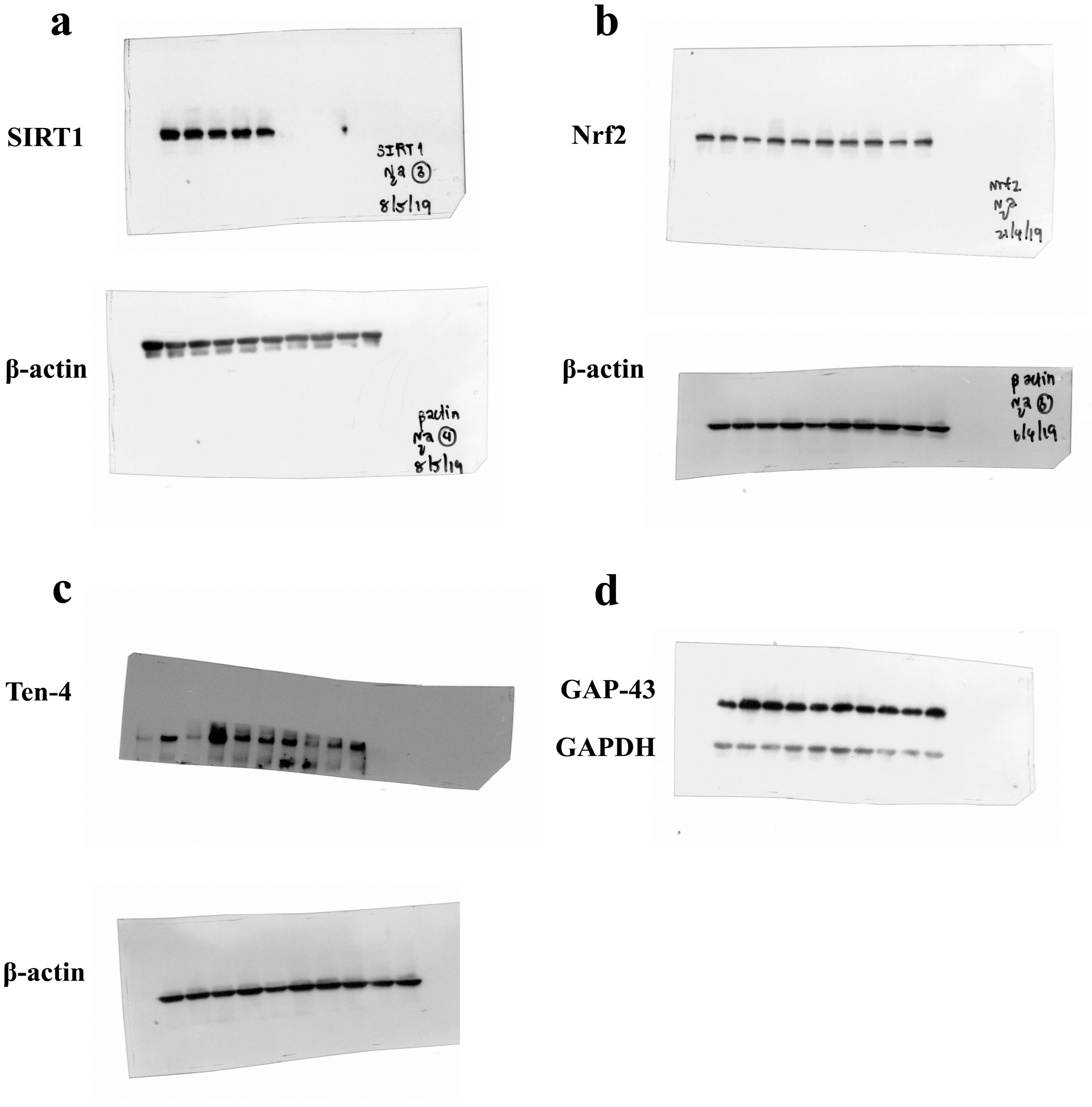

Figure S2. The full images of electrophoretic blots by WB analysis in Neuro-2a cells.

Figure a represents SIRT1 and β-actin protein expressions (GZM (Re3):band2, GZM (Re2):band3, GZM (Re3):band4, control:band 5). Figure b represents Nrf2 and β-actin protein expressions (GZM (Re3):band6, GZM (Re2):band7, GZM (Re3):band8, control:band 9). Figure c represents Ten-4 and β-actin protein expressions (1%FBS:band1, GZM (Re3):band5, GZM (Re2):band6, GZM (Re3);band8. Figure d represents GAP-43 and GAPDH protein expressions (1%FBS:band5, GZM (Re1):band6, GZM (Re2);band7, GZM (Re3);band8.

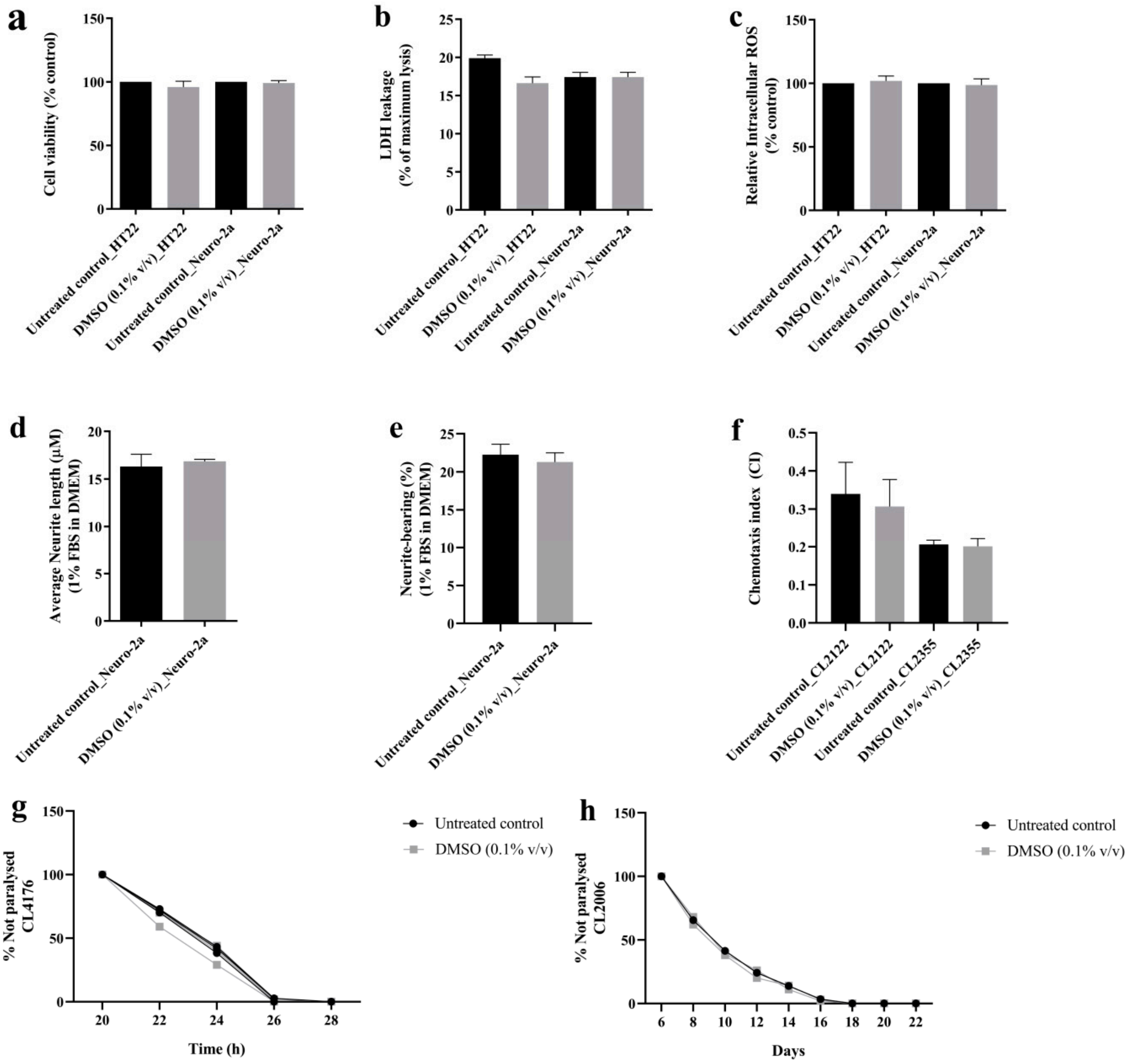

Figure S3. The comparison between untreated control and DMSO (0.1% v/v) control.

There are no significant different between DMSO (0.1% v/v) and untreated control in cell survival (MTT (a), LDH (b)), intracellular ROS (c), neurite outgrowth (neurite length (d), neurite-bearing cells (e)), chemotaxis (f) and paralysis (CL4176 (g), CL2122 (h)) assays.

Table S1. PT50 values for the treatments in paralysis assay

Treatment	PT50 ± SEM	*p*-Value	Significance	Total number
**CL4176**	**(h)**			
Untreated control	23.42 ± 0.13			146
GZM 1.25 µg/ml	25.12 ± 0.05	<0.0001	****	116
GZM 2.5 µg/ml	25.17 ± 0.06	<0.0001	****	124
GZM 5 µg/ml	26.36 ± 0.98	<0.0001	****	97
**CL2006**	**(days)**			
Untreated control	9.30 ± 0.10			87
GZM 1.25 µg/ml	11.60 ± 0.25	<0.0001	****	87
GZM 2.5 µg/ml	11.52 ± 0.13	<0.0001	****	93
GZM 5 µg/ml	14.12 ± 0.07	<0.0001	****	84

Significance was tested compared with the untreated control by one-way ANOVA followed by Bonferroni's method (post-hoc) test, ****$p < 0.0001$

Table S2. The summary of statistical values of glutamate, H_2O_2 , and GZM treatment in neuronal (HT-22 and Neuro-2a) cells

Cells	Treatment	Min	Max	Mean	SD	q	Significant (vs control)	n
		Cell viability(% control)						
N2a	1 h							
	Glutamate 2.5 mM	78.61	88.59	83.06	5.08	4.33	**	3
	Glutamate 5 mM	82.67	91.72	88.35	4.94	2.98	*	3
	Glutamate 10 mM	78.24	90.73	83.52	6.46	4.21	**	3
	6 h							
	Glutamate 2.5 mM	82.28	88.31	85.47	3.03	3.76	*	3
	Glutamate 5 mM	83.88	95.92	89.27	6.11	2.78	*	3
	Glutamate 10 mM	77.92	89.32	85.48	6.55	3.76	*	3
	12 h							
	Glutamate 2.5 mM	62.82	84.42	75.00	11.06	4.10	**	3
	Glutamate 5 mM	73.98	89.59	82.63	7.94	2.85	*	3
	Glutamate 10 mM	71.59	83.82	77.96	6.14	3.61	*	3
	18 h							
	Glutamate 2.5 mM	83.75	92.08	88.81	4.44	2.61	*	3
	Glutamate 5 mM	69.41	79.97	76.38	6.04	5.51	**	3
	Glutamate 10 mM	63.20	77.00	71.55	7.34	6.64	***	3
	24 h							
	Glutamate 2.5 mM	65.82	72.58	68.55	3.56	7.30	****	3
	Glutamate 5 mM	65.46	74.83	69.23	4.94	7.14	****	3
	Glutamate 10 mM	48.39	60.27	54.74	5.98	10.50	****	3
	5 min							
	H_2O_2 100 μM	78.83	111.88	96.66	16.68	0.44	ns	3
	H_2O_2 200 μM	76.27	89.73	83.41	6.77	2.19	ns	3
	H_2O_2 400 μM	91.55	100.20	95.37	4.41	0.61	ns	3
	15 min							
	H_2O_2 100 μM	77.72	98.47	88.42	10.39	39.68	****	3
	H_2O_2 200 μM	73.53	80.92	78.37	4.19	42.84	****	3
	H_2O_2 400 μM	51.45	52.64	52.22	0.66	63.09	****	3
	30 min							
	H_2O_2 100 μM	84.19	88.66	86.50	2.24	5.77	**	3
	H_2O_2 200 μM	72.16	80.59	76.56	4.23	10.03	****	3
	H_2O_2 400 μM	50.33	56.60	53.65	3.15	19.82	****	3
	45 min							
	H_2O_2 100 μM	78.19	89.00	83.26	5.43	6.47	***	3
	H_2O_2 200 μM	47.53	52.46	50.60	2.68	19.10	****	3
	H_2O_2 400 μM	31.99	35.21	34.13	1.85	25.47	****	3
	60 min							
	H_2O_2 100 μM	72.64	78.02	75.93	2.88	19.53	****	3
	H_2O_2 200 μM	47.86	49.22	48.71	0.75	41.61	****	3
	H_2O_2 400 μM	27.57	28.43	27.86	0.50	58.53	****	3
	90 min							

	H_2O_2 100 µM	17.97	21.65	20.39	2.10	70.38	****	3
	H_2O_2 200 µM	19.82	22.39	21.26	1.31	69.62	****	3
	H_2O_2 400 µM	21.53	23.91	22.94	1.25	68.13	****	3
HT22	1 h							
	Glutamate 2.5 mM	85.88	93.12	88.57	3.96	3.96	**	3
	Glutamate 5 mM	78.81	85.09	81.00	3.55	6.58	****	3
	Glutamate 10 mM	82.95	88.82	85.44	3.04	5.04	**	3
	6 h							
	Glutamate 2.5 mM	70.44	93.25	84.73	12.45	2.09	*	3
	Glutamate 5 mM	71.34	77.90	74.86	3.31	3.44	*	3
	Glutamate 10 mM	61.39	91.11	77.50	15.01	3.07	*	3
	12 h							
	Glutamate 2.5 mM	62.40	86.37	77.56	13.18	2.27	*	3
	Glutamate 5 mM	48.76	81.57	65.26	16.40	3.51	*	3
	Glutamate 10 mM	53.15	67.84	62.41	8.06	3.80	*	3
	18 h							
	Glutamate 2.5 mM	70.57	102.30	86.44	22.43	117.90	****	3
	Glutamate 5 mM	54.60	66.40	61.86	6.35	554.90	****	3
	Glutamate 10 mM	36.95	51.53	43.36	7.45	1021.00	****	3
	24 h							
	Glutamate 2.5 mM	33.96	40.06	37.55	3.19	18.33	****	3
	Glutamate 5 mM	28.14	31.01	29.60	1.44	20.67	****	3
	Glutamate 10 mM	13.69	15.02	14.32	0.67	25.15	****	3
	5 min							
	H_2O_2 100 µM	81.78	88.91	85.84	3.67	6.41	***	3
	H_2O_2 200 µM	65.48	69.00	67.44	1.79	14.74	****	3
	H_2O_2 400 µM	60.14	67.24	63.67	3.55	16.45	****	3
	15 min							
	H_2O_2 100 µM	54.88	58.05	56.59	1.60	45.16	****	3
	H_2O_2 200 µM	50.73	51.11	50.89	0.20	51.10	****	3
	H_2O_2 400 µM	45.02	48.36	46.91	1.71	55.24	****	3
	30 min							
	H_2O_2 100 µM	57.58	62.15	59.56	2.35	38.42	****	3
	H_2O_2 200 µM	39.66	41.63	40.45	1.04	56.57	****	3
	H_2O_2 400 µM	21.25	21.75	21.57	0.28	74.51	****	3
	45 min							
	H_2O_2 100 µM	56.04	58.06	57.27	1.07	72.93	****	3
	H_2O_2 200 µM	24.95	26.37	25.88	0.80	126.50	****	3
	H_2O_2 400 µM	12.35	13.24	12.93	0.50	148.60	****	3
	60 min							
	H_2O_2 100 µM	67.25	69.69	68.01	1.46	42.20	****	3
	H_2O_2 200 µM	16.77	18.46	17.84	0.93	108.40	****	3
	H_2O_2 400 µM	14.85	16.07	15.63	0.68	111.30	****	3
	90 min							
	H_2O_2 100 µM	30.85	35.81	33.83	2.62	49.56	****	3
	H_2O_2 200 µM	11.63	13.56	12.71	0.98	65.37	****	3

	H_2O_2 400 μM	12.30	15.60	14.14	1.68	64.30	****	3
N2a								
	GZM 0.5 μg/ml	89.68	100.41	95.93	4.61	2.14	ns	7
	GZM 1 μg/ml	94.53	100.29	95.96	4.97	2.12	ns	7
	GZM 10 μg/ml	84.30	99.78	92.17	6.46	4.11	ns	7
HT22								
	GZM 0.5 μg/ml	96.42	120.82	105.10	8.10	1.96	ns	7
	GZM 1 μg/ml	89.67	109.23	99.34	6.41	0.25	ns	7
	GZM 10 μg/ml	96.84	112.30	104.22	6.01	1.62	ns	7

NS, $p \geq 0.05$, *$p < 0.05$, **$p < 0.01$, ***$p < 0.001$ and ****$p < 0.0001$ compared with the control.

Table S3. The summary of statistical values of H_2O_2 and GZM extract treatment in neuronal (HT-22 and Neuro-2a) cells

Cells	Treatment	Min	Max	Mean	SD	q	Significant (vs H_2O_2 control)	n
		Cell viability(% control)						
N2a	DMSO control	51.69	55.73	53.67	1.51	23.16	****	6
	H_2O_2 400 μM	60.82	72.85	66.78	5.05			6
	H_2O_2 400 μM GZM 0.5 μg/ml	61.31	68.04	64.22	2.41	5.84	****	6
	H_2O_2 400 μM GZM 1 μg/ml	59.78	66.72	63.18	2.44	5.26	***	4
	H_2O_2 400 μM GZM 10 μg/ml	51.69	55.73	53.67	1.51	23.16	****	6
HT22	DMSO control	52.50	56.55	54.02	1.46	45.65	****	3
	H_2O_2 400 μM	85.80	87.11	87.14	1.36			3
	H_2O_2 200 μM GZM 0.5 μg/ml	81.10	84.41	82.82	1.66	25.74	****	3
	H_2O_2 200 μM GZM 1 μg/ml	82.15	87.17	84.60	2.51	27.35	****	4
	H_2O_2 200 μM GZM 10 μg/ml	52.50	56.55	54.02	1.46	45.65	****	3
		LDH leakage (% of maximum lysis)						
N2a	DMSO control	16.74	17.96	17.50	0.55	19.68	****	7
	H_2O_2 400 μM	51.81	68.34	58.74	8.59			7
	H_2O_2 200 μM GZM 0.5 μg/ml	21.20	23.39	22.58	1.20	14.06	****	3
	H_2O_2 200 μM GZM 1 μg/ml	21.31	23.51	22.69	1.20	14.02	****	3
	H_2O_2 200 μM GZM 10 μg/ml	21.49	23.75	22.91	1.23	13.94	****	3
HT22	DMSO control	15.77	20.40	17.92	1.96	17.91	****	9
	H_2O_2 200 μM	53.67	69.79	60.74	8.24			9
	H_2O_2 200 μM GZM 0.5 μg/ml	23.70	25.97	25.13	1.25	12.59	****	3
	H_2O_2 200 μM GZM 1 μg/ml	23.95	25.95	25.38	1.25	12.50	****	3
	H_2O_2 200 μM GZM 10 μg/ml	24.60	26.87	26.00	1.22	12.28	****	3

NS, $p \geq 0.05$, *$p < 0.05$, **$p < 0.01$, ***$p < 0.001$ and ****$p < 0.0001$ compared with the control.

Table S4. The summary of statistical values of glutamate and GZM extract treatment in neuronal (HT-22 and Neuro-2a) cells

Cells	Treatment	Min	Max	Mean	SD	q	Significant (vs glutamate control)	n
		Cell viability(% control)						
N2a	DMSO control	55.02	65.89	60.70	3.92	12.15	****	6
	Glutamate 10 mM	77.95	91.98	84.42	5.57			6
	Glutamate 10 mM GZM 0.5 μg/ml	83.77	100.21	91.77	7.02	10.64	****	6
	Glutamate 10 mM GZM 1 μg/ml	93.40	100.48	98.79	3.11	13.05	****	4
	Glutamate 10 mM GZM 10 μg/ml	55.02	65.89	60.70	3.92	12.15	****	6
HT22	DMSO control	54.00	57.17	55.29	1.18	28.74	****	6
	Glutamate 5 mM	74.61	83.13	78.75	3.72			6
	Glutamate 5 mM GZM 0.5 μg/ml	71.37	79.57	74.18	2.91	13.57	****	3
	Glutamate 5 mM GZM 1 μg/ml	73.39	76.62	75.50	1.82	11.72	****	4
	Glutamate 5 mM GZM 10 μg/ml	54.00	57.17	55.29	1.18	28.74	****	6
		LDH leakage (% of maximum lysis)						
N2a	DMSO control	15.77	20.40	17.92	1.96	19.43	****	7
	Glutamate 10 mM	53.67	69.79	60.74	8.24			7
	Glutamate 10 mM GZM 0.5 μg/ml	25.79	30.25	28.24	2.27	11.99	****	3
	Glutamate 10 mM GZM 1 μg/ml	26.13	29.07	27.65	1.47	12.21	****	3
	Glutamate 10 mM GZM 10 μg/ml	29.01	30.72	29.68	0.91	11.46	****	3
HT22	DMSO control	17.01	19.70	18.89	1.30	17.20	****	9
	Glutamate 5 mM	54.84	70.00	63.69	7.89			9
	Glutamate 5 mM GZM 0.5 μg/ml	25.04	26.96	26.39	1.17	12.10	****	3
	Glutamate 5 mM GZM 1 μg/ml	25.33	31.39	27.38	3.48	11.78	****	3
	Glutamate 5 mM GZM 10 μg/ml	25.52	33.17	28.14	4.36	11.53	****	3

NS, $p \geq 0.05$, *$p < 0.05$, **$p < 0.01$, ***$p < 0.001$ and ****$p < 0.0001$ compared with the control.

Table S5. The summary of statistical values of intracellular ROS levels and antioxidant enzyme genes expression after GZM extract treatment in neuronal (HT-22 and Neuro-2a) cells

Cells	Treatment	Min	Max	Mean	SD	q	Significant (vs glutamate control)	n
		Relative intracellular ROS (%control)						
N2a	DMSO control	185.74	200.00	192.32	7.19	24.95	****	3
	Glutamate 10 mM	109.34	121.24	115.16	5.95			3
	Glutamate 10 mM GZM 0.5 μg/ml	112.43	120.84	116.37	4.23	19.20	****	3
	Glutamate 10 mM GZM 1 μg/ml	129.05	136.06	132.55	4.96	13.51	****	3
	Glutamate 10 mM GZM 10 μg/ml	185.74	200.00	192.32	7.19	24.95	****	3
HT22	DMSO control	161.11	184.83	175.77	11.00	13.24	****	6
	Glutamate 5 mM	112.47	132.39	121.21	10.18			3
	Glutamate 5 mM GZM 0.5 μg/ml	109.35	120.61	116.17	5.99	9.57	****	3
	Glutamate 5 mM GZM 1 μg/ml	106.07	124.92	116.46	9.57	9.52	****	3
	Glutamate 5 mM GZM 10 μg/ml	161.11	184.83	175.77	11.00	13.24	****	3
		(Fold change)					Significant (vs control)	
N2a								4
GSTa2	DMSO control	0.76	1.15	0.95	0.18			4
	GZM 10 μg/ml	1.44	1.65	1.55	0.15	13.63	****	4
GSTo1	DMSO control	0.81	1.08	0.97	0.12			4
	GZM 10 μg/ml	1.66	2.07	1.86	0.29	5.39	***	4
GPX	DMSO control	1.01	1.28	1.14	0.16			4
	GZM 10 μg/ml	1.40	1.78	1.60	0.17	6.54	***	4
CAT	DMSO control	0.93	1.41	1.17	0.26			4
	GZM 10 μg/ml	0.88	1.25	1.03	0.16	1.86	NS	4
SOD1	DMSO control	0.93	1.07	1.08	0.16			4
	GZM 10 μg/ml	1.92	2.08	2.00	0.08	4.85	***	4
SOD2	DMSO control	0.71	1.03	0.88	0.16			4
	GZM 10 μg/ml	1.74	2.21	1.97	0.34	4.63	**	4
HT22								4
GSTa2	DMSO control	0.76	1.15	0.95	0.18			4
	GZM 10 μg/ml	1.44	1.65	1.55	0.15	4.46	**	4
GSTo1	DMSO control	0.81	1.08	0.97	0.12			4
	GZM 10 μg/ml	1.66	2.07	1.86	0.29	4.82	**	4
GPX	DMSO control	0.99	1.28	1.14	0.16			4

	GZM 10 μg/ml	1.40	1.78	1.60	0.17	5.04	**	4
CAT	DMSO control	0.93	1.41	1.17	0.26			4
	GZM 10 μg/ml	0.88	1.25	1.03	0.16	0.11	NS	4
SOD1	DMSO control	0.93	1.30	1.08	0.16			4
	GZM 10 μg/ml	1.92	2.08	2.00	0.08	9.03	****	4
SOD2	DMSO control	0.71	1.03	0.88	0.16			4
	GZM 10 μg/ml	1.74	2.21	1.97	0.34	4.42	**	4

NS, $p \geq 0.05$, *$p < 0.05$, **$p < 0.01$, ***$p < 0.001$ and ****$p < 0.0001$ compared with the control.

Table S6. The summary of statistical values of SIRT-1 and Nrf2 expression after GZM extract treatment in Neuro-2a cells

Cells	Treatment	Min	Max	Mean	SD	q	Significant (vs control)	n
		Relative protein expression (Fold change)						
N2a								
SIRT-1	DMSO control							6
	Glutamate 10 mM	1.16	1.73	1.52	0.30	2.90	*	6
N2a								
Nrf2	DMSO control							4
	Glutamate 10 mM	1.31	1.82	1.60	0.27	4.50	**	4
		(Fold change)						
N2a								
Nqo1	DMSO control	0.75	1.21	0.98	0.25			6
	GZM 10 μg/ml	1.13	1.85	1.54	0.35	3.72	**	6
Gclm	DMSO control	0.95	1.16	1.05	0.08			6
	GZM 10 μg/ml	1.36	1.43	1.36	0.06	5.30	***	6
EAAT3	DMSO control	0.78	1.16	0.99	0.21			6
	GZM 10 μg/ml	1.49	2.05	1.68	0.26	5.23	***	6
SIRT-1	DMSO control	0.93	1.07	0.90	0.19			6
	GZM 10 μg/ml	1.45	1.56	1.50	0.06	7.39	****	6
HT22								
Nqo1	DMSO control	0.72	1.06	0.88	0.14			6
	GZM 10 μg/ml	1.44	2.10	1.70	0.31	1.15	***	6
Gclm	DMSO control	0.84	1.09	0.95	0.10			6
	GZM 10 μg/ml	1.64	1.96	1.79	0.17	5.16	***	6
EAAT3	DMSO control	0.64	1.20	0.96	0.19			6
	GZM 10 μg/ml	1.24	2.03	1.59	0.33	3.56	**	6
SIRT-1	DMSO control	0.87	1.30	1.10	0.21			6
	GZM 10 μg/ml	1.23	1.88	1.64	0.25	2.99	*	6

NS, $p \geq 0.05$, *$p < 0.05$, **$p < 0.01$, ***$p < 0.001$ and ****$p < 0.0001$ compared with the control.

Table S7. The summary of statistical values of neurite outgrowth after GZM extract treatment in Neuro-2a cells

Cells	Treatment	Min	Max	Mean	SD	q	Significant (vs control)	n
		Neurite length (μM)						
N2a	10% FBS in DMEM	5.60	7.50	6.66	0.97	10.56	****	3
	1% FBS in DMEM (control)	13.74	17.89	16.30	8.35			3
	GZM 10 μg/ml	29.28	32.55	30.92	1.63	17.52	****	3
		Neurite bearing cells (%)						
N2a	10% FBS in DMEM	3.85	4.00	4.44	1.30	20.78	****	6
	1% FBS in DMEM (control)	20.61	23.97	22.26	1.38			5
	GZM 10 μg/ml	49.14	57.00	52.35	3.23	12.79	****	6
		mRNA expression (fold change)						
N2a	GAP43							
	1% FBS in DMEM (control)	1.50	1.71	1.59	0.80			3
	GZM 10 μg/ml	3.76	3.69	3.73	0.05	39.78	****	3
		protein expression (fold change)						
N2a	GAP43							
	1% FBS in DMEM (control)	1	1	1				4
	GZM 10 μg/ml	2.41	3.40	2.92	0.43	8.89	***	4
		mRNA expression (fold change)						
N2a	Ten-4							
	1% FBS in DMEM (control)	1.68	1.82	2.43	0.81			1 2
	GZM 10 μg/ml	3.34	5.03	4.35	0.76	6.24	****	
		protein expression (fold change)						
N2a	Ten-4							
	1% FBS in DMEM (control)	1	1	1				3
	GZM 10 μg/ml	2.27	3.18	2.86	0.51	7.53	***	3

NS, $p \geq 0.05$, *$p < 0.05$, **$p < 0.01$, ***$p < 0.001$ and ****$p < 0.0001$ compared with the control.

Table S8. The summary of statistical values of neuroprotective effect after GZM extract treatment in *C. elegans*

Strain	Treatment	Min	Max	Mean	SD	q	Significant (vs control)	n
		% Not paralysed (24 h)						
CL4176	DMSO control	38.30	43.24	41.53	2.23			60
	GZM 1.5 µg/ml	83.87	87.18	85.74	1.39	0.73	****	62
	GZM 2.5 µg/ml	85.00	91.30	88.95	2.96	0.75	****	65
	GZM 5 µg/ml	75.68	79.38	76.70	1.79	0.65	****	69
		% Not paralysed (12 days)						
CL2006	DMSO control	22.14	26.15	24.14	0.21			63
	GZM 1.5 µg/ml	35.93	38.78	37.93	0.06	1.48	****	70
	GZM 2.5 µg/ml	39.94	44.84	41.94	0.26	4.24	****	64
	GZM 5 µg/ml	73.56	78.31	75.00	5.05	6.33	****	72
		Chemotaxis index (CI)						
CL2355	DMSO control	0.19	0.22	0.20	0.01			66
	GZM 5 µg/ml	0.38	0.48	0.43	0.04	0.95	*	68
CL2122	DMSO control	0.24	0.39	0.34	0.07			63
	GZM 5 µg/ml	0.32	0.35	0.33	0.01	0.08	NS	65

NS, $p \geq 0.05$, *$p < 0.05$, **$p < 0.01$, ***$p < 0.001$ and ****$p < 0.0001$ compared with the control.
