# Supplementary figures and images for: Neuroprotective Effects of Glochidion zeylanicum Leaf Extract against H2O2/Glutamate-Induced Toxicity in Cultured Neuronal Cells and Aβ-Induced Toxicity in Caenorhabditis elegans
Source: Biology (Basel). 2021 Aug 19;10(8):800. doi: 10.3390/biology10080800 (PMC8389654; doi:10.3390/biology10080800)

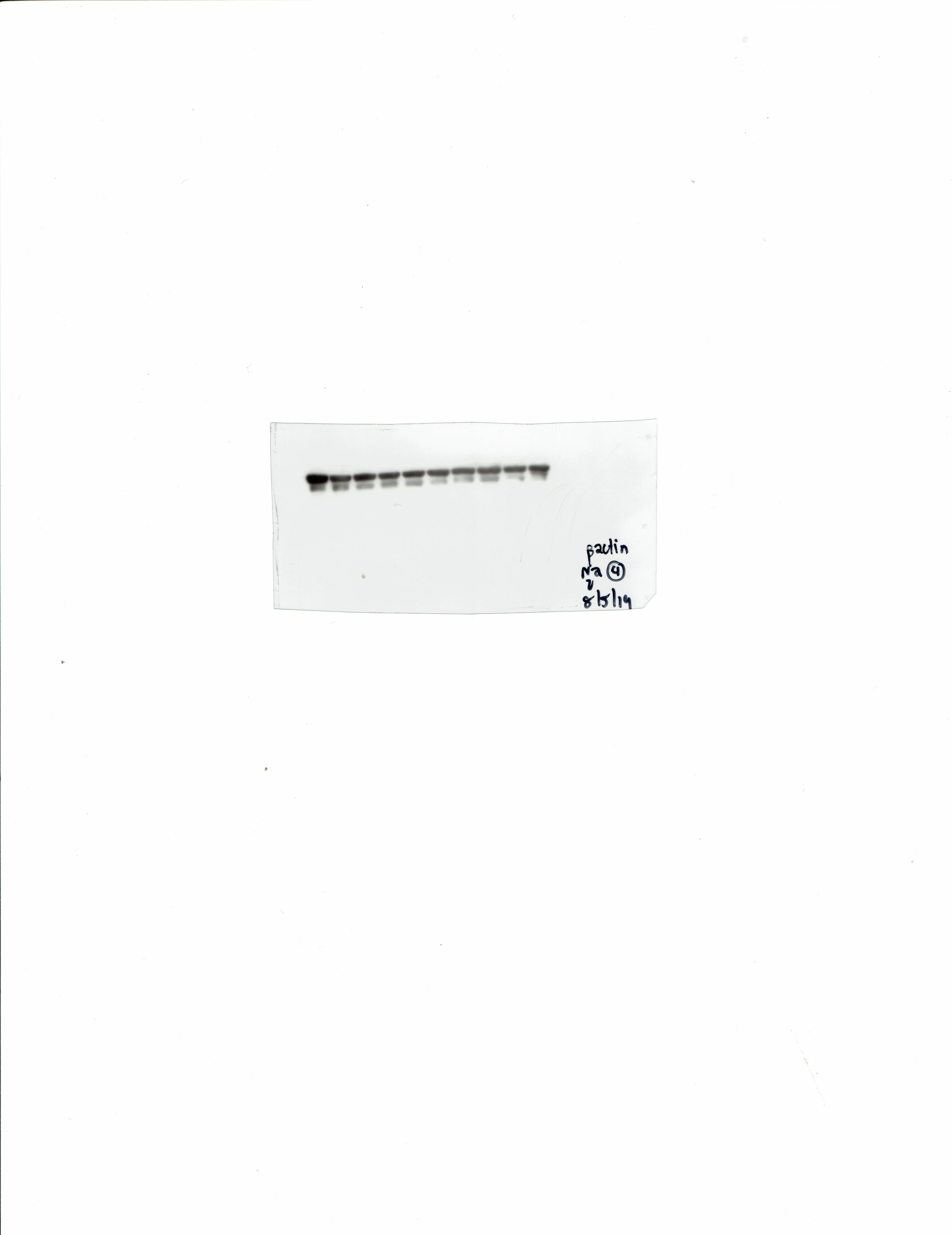

Supplement: Supplementary file 1 [file biology-10-00800-s001.zip › WB/S2a_N2a_Bactin.jpg]

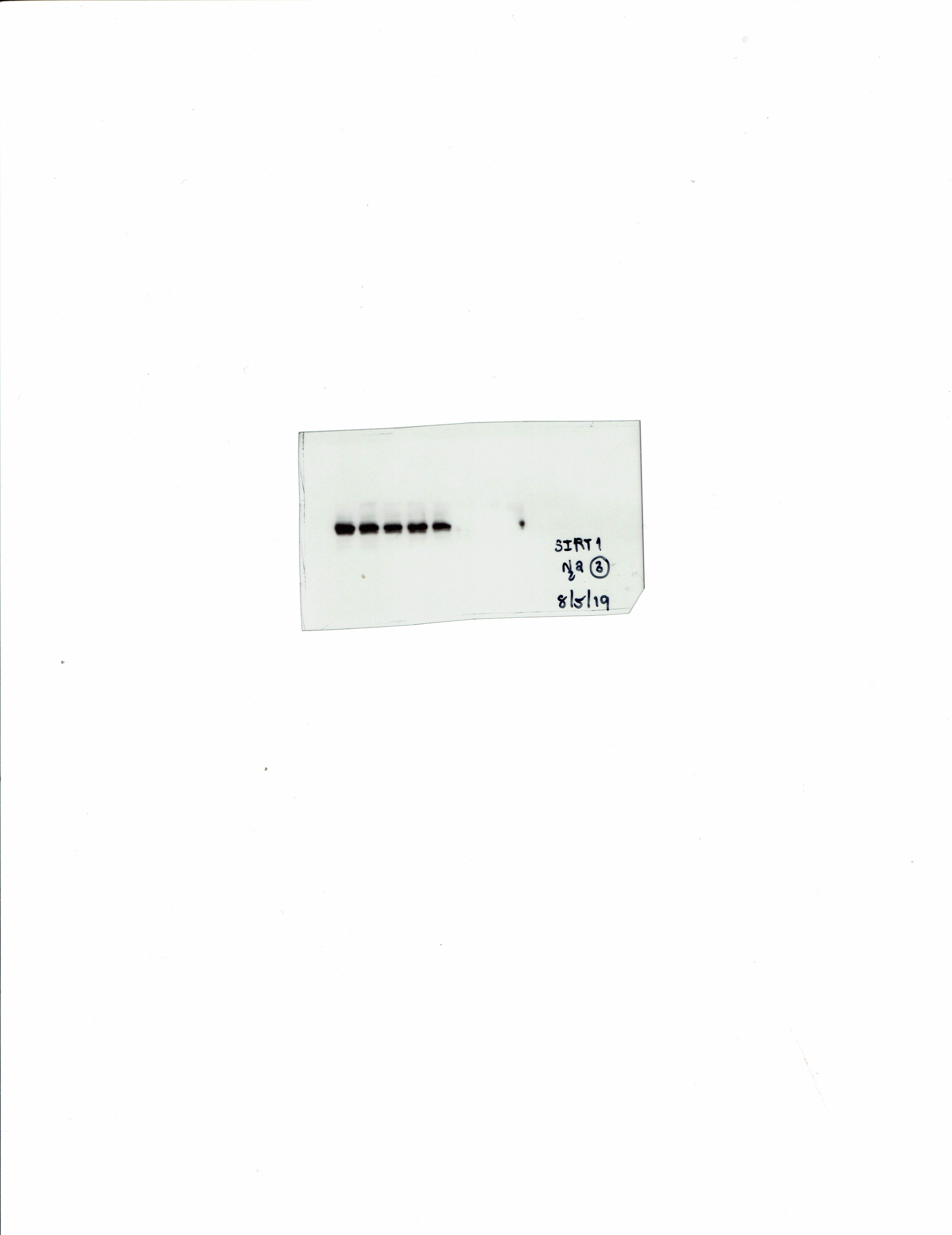

Supplement: Supplementary file 1 [file biology-10-00800-s001.zip › WB/S2a_N2a_Sirt1_.jpg]

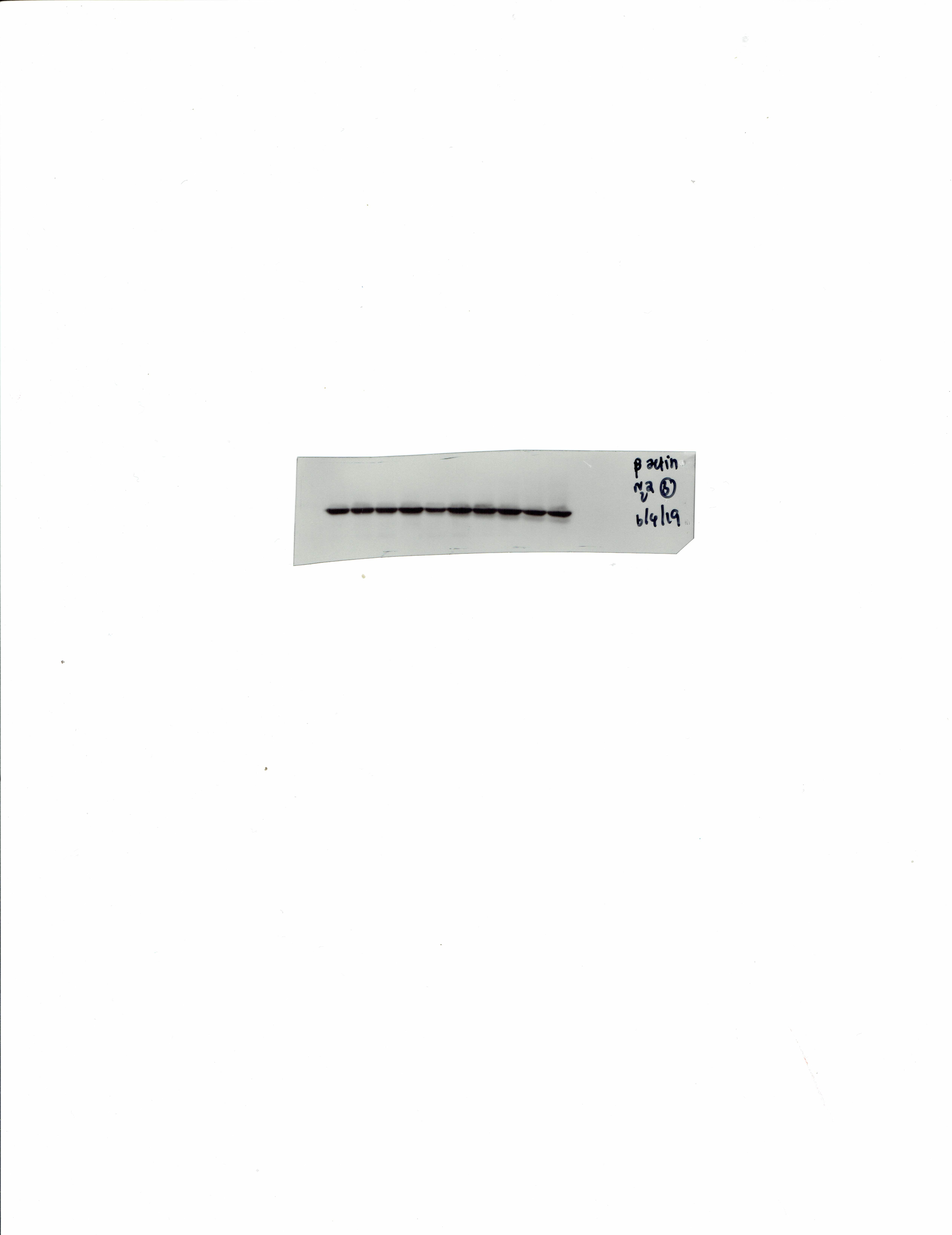

Supplement: Supplementary file 1 [file biology-10-00800-s001.zip › WB/S2b_N2a_Bactin.jpg]

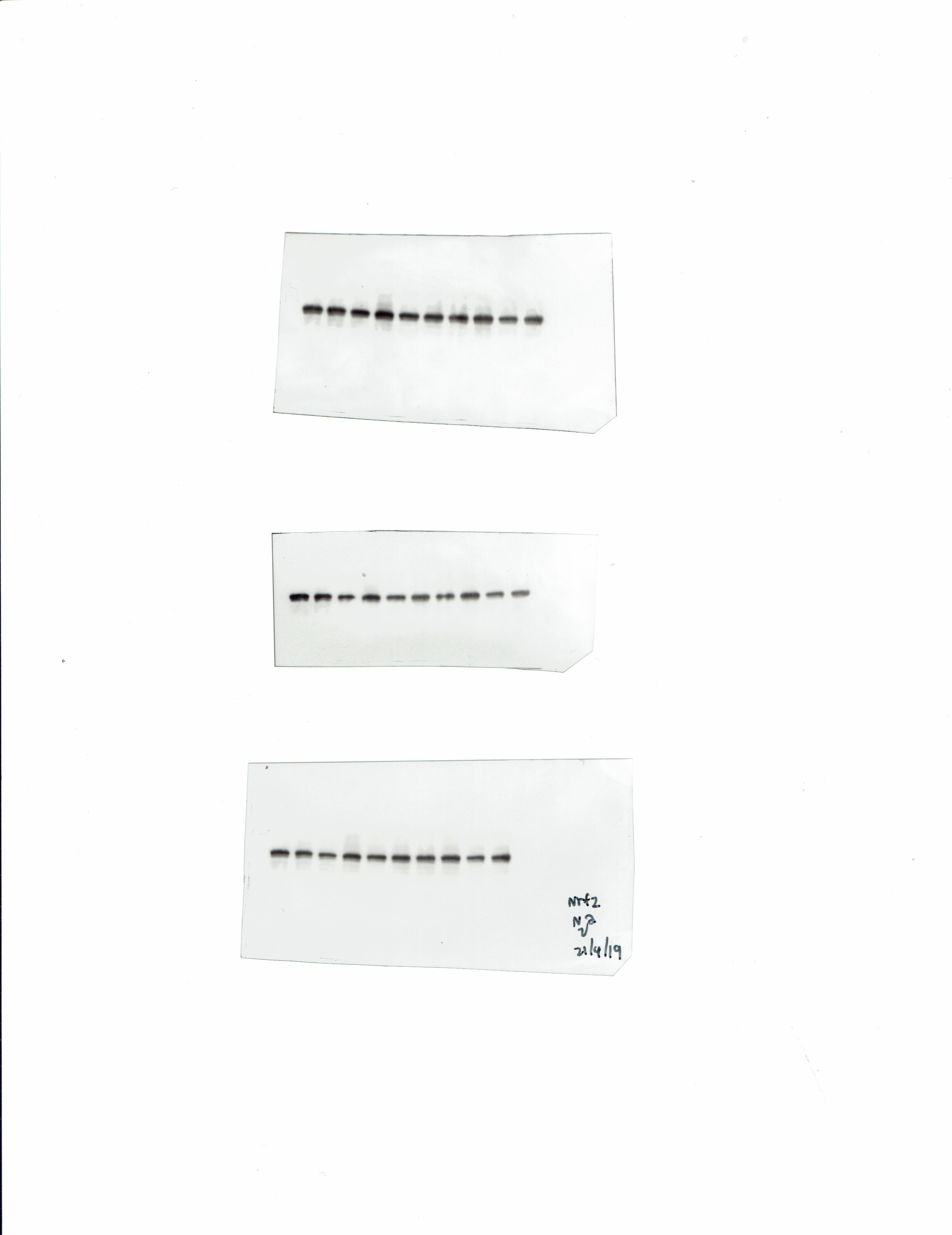

Supplement: Supplementary file 1 [file biology-10-00800-s001.zip › WB/S2b_N2a_Nrf22.jpg]

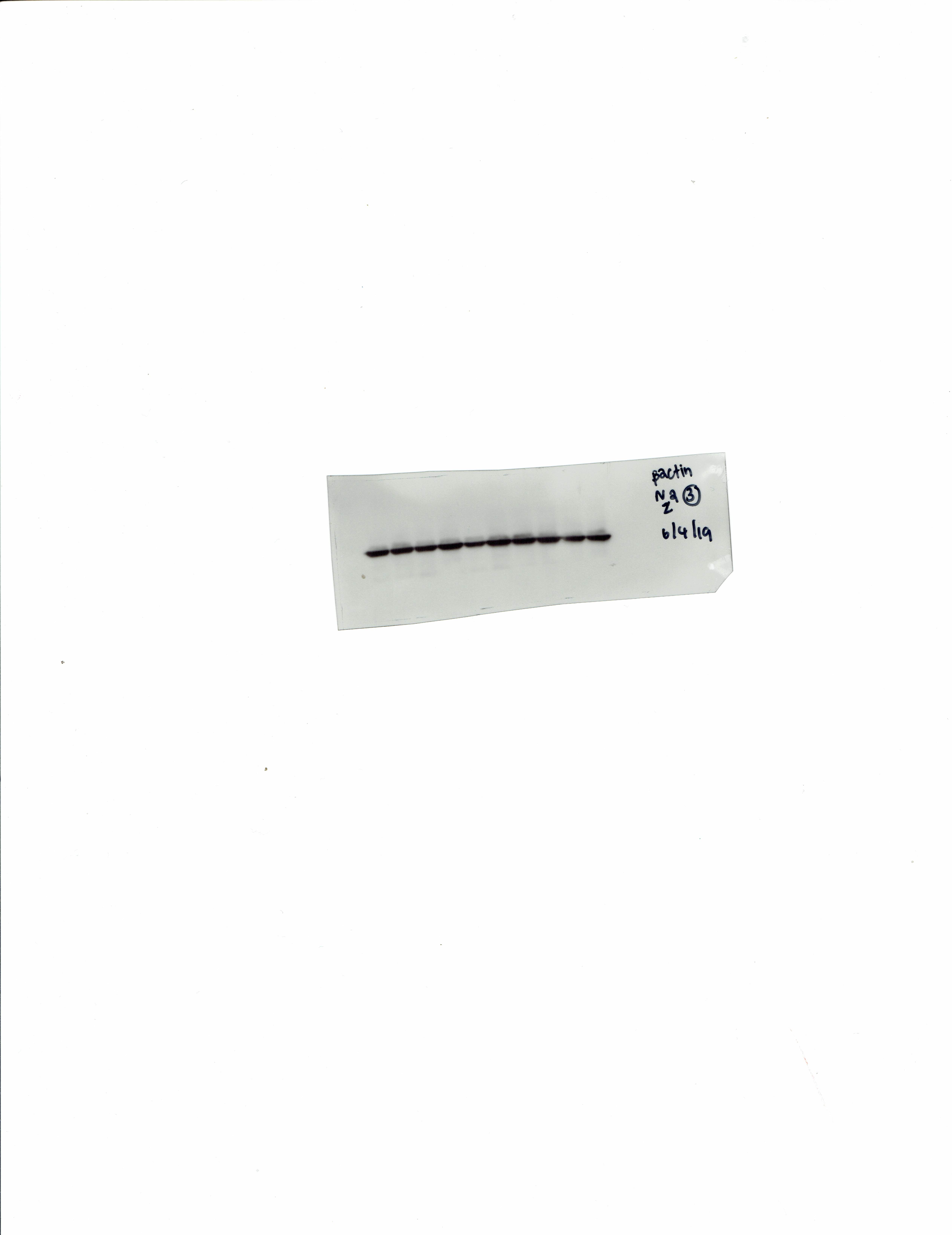

Supplement: Supplementary file 1 [file biology-10-00800-s001.zip › WB/S2c_N2a_Bactin.jpg]

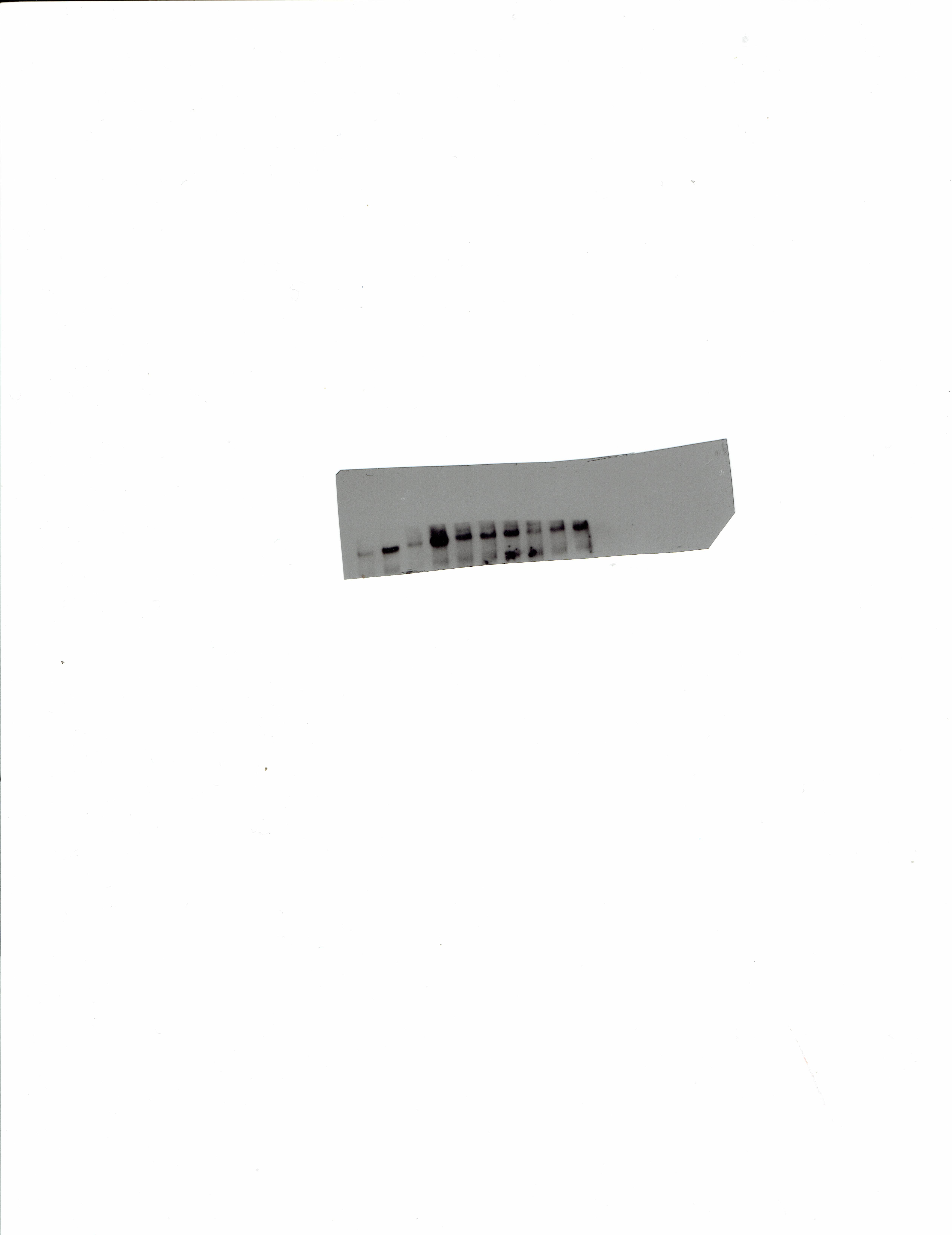

Supplement: Supplementary file 1 [file biology-10-00800-s001.zip › WB/S2c_N2a_Ten4.jpg]

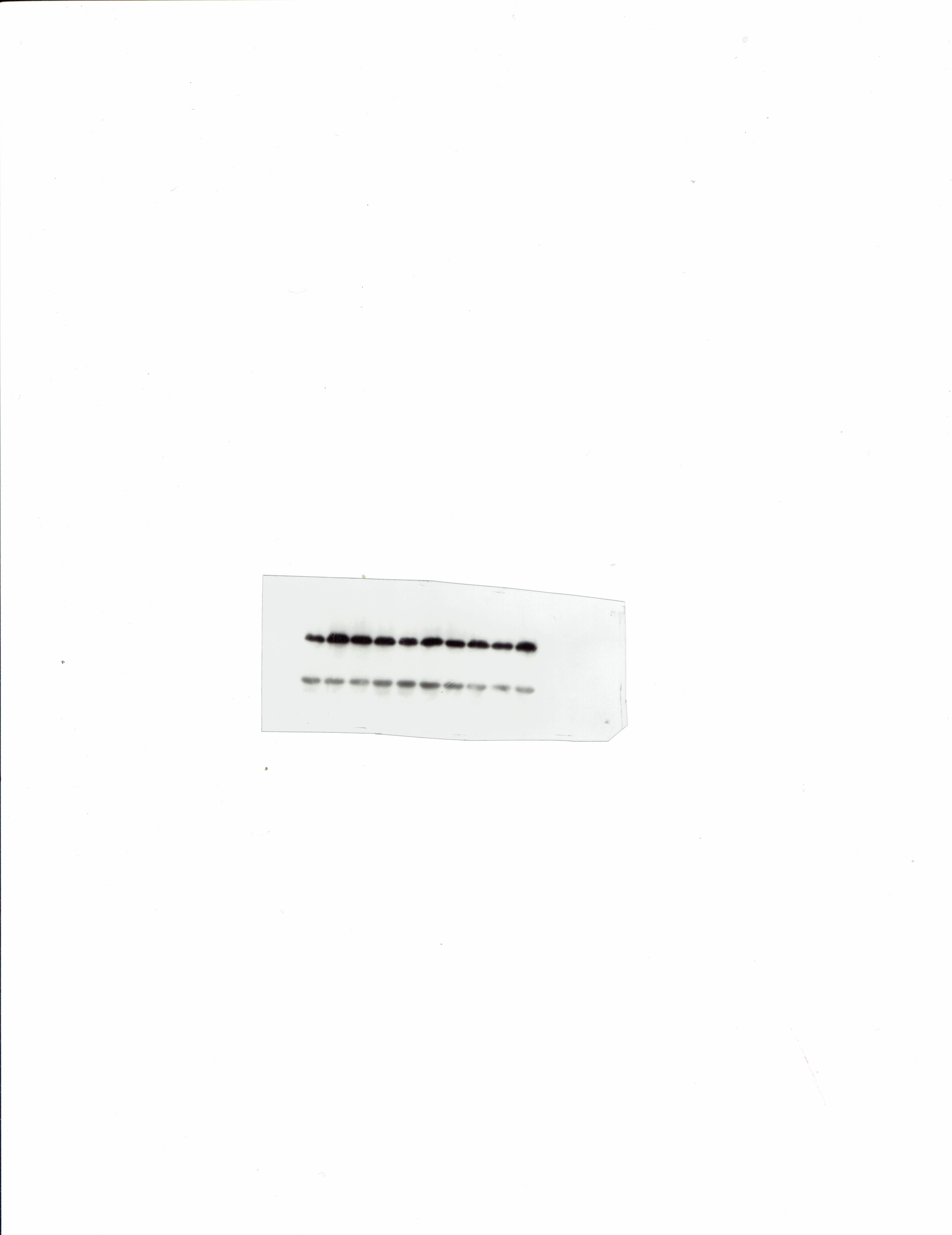

Supplement: Supplementary file 1 [file biology-10-00800-s001.zip › WB/S2d_N2a_GAP43_GAPDH2_.jpg]
